# Supplementary material for: Transplantation of high-risk donor livers after resuscitation and viability assessment using a combined protocol of oxygenated hypothermic, rewarming and normothermic machine perfusion: study protocol for a prospective, single-arm study (DHOPE-COR-NMP trial)
Source: BMJ Open. 2019 Aug 15;9(8):e028596. doi: 10.1136/bmjopen-2018-028596 (PMC6701560; doi:10.1136/bmjopen-2018-028596)
Supplement: Author's manuscript [file bmjopen-2018-028596.draft_revisions.pdf]

BMJ Open is committed to open peer review. As part of this commitment we make the peer review history of every article we publish publicly available.

When an article is published we post the peer reviewers' comments and the authors' responses online. We also post the versions of the paper that were used during peer review. These are the versions that the peer review comments apply to.

The versions of the paper that follow are the versions that were submitted during the peer review process. They are not the versions of record or the final published versions. They should not be cited or distributed as the published version of this manuscript.

BMJ Open is an open access journal and the full, final, typeset and author-corrected version of record of the manuscript is available on our site with no access controls, subscription charges or pay-per-view fees (http://bmjopen.bmj.com).

If you have any questions on BMJ Open's open peer review process please email info.bmjopen@bmj.com

BMJ Open

Transplantation of high-risk donor livers after resuscitation and viability assessment using a combined protocol of oxygenated hypothermic, rewarming and normothermic machine perfusion: a prospective, single arm study DHOPE – COR – NMP Trial

Journal:	*BMJ Open*
Manuscript ID	bmjopen-2018-028596
Article Type:	Protocol
Date Submitted by the Author:	18-Dec-2018

Complete List of Authors:	de Vries, Yvonne; University Medical Center Groningen, Hepatobiliary surgery and Liver Transplantation<br>Berendsen, Tim ; Hepatobiliary Surgery and Liver Transplantation<br>Fujiyoshi, Masato; Hepatobiliary Surgery and Liver Transplantation<br>van den Berg, Aad; Gasteroenterology and Hepatology<br>Blokzijl, Hans; Universitair Medisch Centrum Groningen, Gastroenterology and Hepatology<br>de Boer, Marieke; Hepatobiliary Surgery and Liver Transplantation<br>van der Heide , Frans; Gasteroenterology and Hepatology<br>de Kleine, Ruben; Hepatobiliary Surgery and Liver Transplantation<br>van Leeuwen, Otto; Hepatobiliary Surgery and Liver Transplantation<br>Matton, Alix; Hepatobiliary Surgery and Liver Transplantation<br>Werner, Maureen; Hepatobiliary Surgery and Liver Transplantation<br>De Meijer, Vincent; Universitair Medisch Centrum Groningen, Surgery<br>Lisman, Ton; Surgical Research Laboratory<br>Porte, Robert; Universitair Medisch Centrum Groningen, Hepatobiliary Surgery and Liver Transplantation

Keywords:	Extended criteria donor liver, Machine perfusion, Liver transplantation, Viability testing, Hemoglobin-based oxygen carrier

SCHOLARONE™
Manuscripts

Transplantation of high-risk donor livers after resuscitation and viability assessment using a combined protocol of oxygenated hypothermic, rewarming and normothermic machine perfusion: a prospective, single arm study

DHOPE – COR – NMP Trial

Yvonne de Vries[1,2], Tim A. Berendsen[1], Masato Fujiyoshi[1], Aad P. van den Berg[3], Hans Blokzijl[3], Marieke T. de Boer[1], Frans van der Heide[3], Ruben H. J. de Kleine[1], Otto B. van Leeuwen[1,2], Alix P. M. Matton[1,2], Maureen J.M. Werner[1], Vincent E. de Meijer[1], Ton Lisman[2], Robert J. Porte[1]

1) Section Hepatobiliary Surgery and Liver Transplantation, Department of Surgery, University of Groningen, University Medical Center Groningen, Groningen, The Netherlands

2) Surgical Research Laboratory, Department of Surgery, University of Groningen, University Medical Center Groningen, Groningen, The Netherlands

3) Department of Gastroenterology and Hepatology, University of Groningen, University Medical Center Groningen, Groningen, The Netherlands

<u>Corresponding author:</u>

Robert J. Porte, MD, PhD

Section of HPB Surgery and Liver Transplantation

Department of Surgery

University Medical Center Groningen

P.O. Box 30.001

9700 RB Groningen

The Netherlands

Telephone: +31-50-3612896

Fax: +31-50-3614873

E-mail: r.j.porte@umcg.nl

Keywords:

- Extended criteria donor liver
- Machine perfusion
- Hemoglobin-based oxygen carrier
- Liver transplantation
- Viability testing

Word count:

Title: 241 characters

Abstract: 293 words

Main text: 3908 words (max. 4000)

For peer review only - http://bmjopen.bmj.com/site/about/guidelines.xhtml

ABSTRACT

Introduction: Extended criteria donor (ECD) livers are increasingly accepted for transplantation in an attempt to reduce the gap between the number of patients on the waiting list and the available number of donor livers. ECD livers, however, carry an increased risk of developing primary non-function (PNF), early allograft dysfunction (EAD) or post-transplant cholangiopathy. Ischemia-reperfusion injury (IRI) plays an important role in the development of these complications. Machine perfusion reduces IRI and allows for reconditioning and subsequent evaluation of liver grafts. Single or dual hypothermic oxygenated machine perfusion (DHOPE) (4 – 12 °C) decreases IRI by resuscitation of mitochondria. Controlled oxygenated rewarming (COR) may further reduce IRI by preventing sudden temperature shifts. Subsequent normothermic machine perfusion (NMP) (37 °C) allows for *ex situ* viability assessment to facilitate the selection of ECD livers with a low risk of PNF, EAD, or post-transplant cholangiopathy.

Methods and analysis: This prospective, single arm study is designed to resuscitate and evaluate initially nationwide declined ECD livers. End-ischemic DHOPE will be performed for initial mitochondrial and graft resuscitation, followed by COR of the donor liver to a normothermic temperature. Subsequently, NMP will be continued to assess viability of the liver. Transplantation into eligible recipients will proceed if all predetermined viability criteria are met within the first 150 min of NMP. To facilitate machine perfusion at different temperatures a perfusion solution containing a haemoglobin-based oxygen carrier (HBOC)-based will be used. With this protocol we aim to transplant extra livers. The primary endpoint is graft survival at 3 months after transplantation.

Ethics and dissemination: This protocol was approved by the medical ethical committee of Groningen, METc2016.281 in August 2016 and registered in the Dutch Trial Register: www.trialregister.nl (NTR5972). Inclusion has been started mid-2017 and is expected to be completed in 2019.

For peer review only - http://bmjopen.bmj.com/site/about/guidelines.xhtml

ARTICLE SUMMARY

Strengths and limitations of this study

- This protocol combines the benefits (resuscitation and *ex situ* viability testing) of multiple machine perfusion modalities (DHOPE, COR and NMP);
- The rewarming phase (COR) avoids sudden temperature shifts that may cause additional injury to an already compromised donor organ;
- A newly developed perfusion fluid containing a hemoglobin-based oxygen carrier allows for perfusion at different temperatures, yet is not officially registered in the Netherlands;
- Initially nationwide declined high-risk donor livers, which carry an increased risk of early graft failure due to PNF, EAD or post-transplant cholangiopathy, will be accepted for this machine perfusion protocol.

INTRODUCTION

Increased use of ECD livers

Driven by the donor organ shortage, extended criteria donor (ECD) or suboptimal quality livers are increasingly used for transplantation. These livers would have been refused for transplantation in the past, because of donor characteristics (advanced age, obesity, alcohol abuse, long ICU stay with use of vasopressors) or graft pathology (steatosis, donation after cardiac death {DCD}, elevated liver enzymes, history of liver trauma), but are nowadays accepted due to the shortage of suitable donor livers. ECD livers carry an increased risk of developing primary non-function (PNF), early allograft dysfunction (EAD), or post-transplant cholangiopathy (1). These complications are difficult to predict, causing potentially transplantable ECD livers to be declined. It has been predicted that in the USA the donor liver discard rate will exceed 50% in the next two decades (2,3). In 2016, in the Netherlands 235 donation procedures were effectuated, yet 75 liver grafts were not used, equaling a discard rate of 32% (4).

Machine perfusion as a tool to resuscitate ECD livers

In previous animal and pre-clinical human liver studies, machine perfusion has been applied as a tool to improve and evaluate donor livers prior to transplantation (5-7). Many machine perfusion protocols exist, with varying temperature, perfusion solution, and other characteristics. Oxygenated hypothermic machine perfusion (HMP), which is performed at 4 – 12 °C, aims to resuscitate the mitochondria and sustain adenosine 5'-triphosphate (ATP) production through the supply of oxygen, while the cold keeps metabolic rates suppressed (8-10). Schlegel et al. have shown that 2 hours of oxygenated HMP is sufficient to replenish cellular energy stores (9). A pilot study by our research group, which included 30 DCD livers, has shown a 100% graft survival in the dual hypothermic oxygenated perfusion (DHOPE)

group (n=10), compared to 70% in the static cold storage (SCS) group (n=20). Furthermore, the incidence of non-anastomotic strictures (NAS) of the biliary tree was 10% in the DHOPE group, compared to 35% in the SCS group (10). Based on these favorable results, our centre initiated a multicentre randomized controlled trial on DHOPE (ClinicalTrial.gov identifier NCT02584283).

Several research groups have investigated the use of mid/subnormothermic machine perfusion or controlled oxygenated rewarming (COR), performed at 12 – 34 °C (8). COR may function as a bridge between hypo- and normothermic machine perfusion, omitting a sudden temperature shift, but may also have an IRI reducing effect (7). An abrupt temperature shift contributes to mitochondrial dysfunction, and is more pronounced during reperfusion after HMP, than after COR (7,11). COR has also been described to increase cellular energy content in a clinical study. Furthermore, in a clinical trial performed by the group in Essen, ECD graft survival was a 100% at 6 months after transplantation in the COR group, whereas this was 85% in the SCS group (7,11).

Machine perfusion as a tool to evaluate ECD livers

A more accurate determination of organ viability can be provided by normothermic machine perfusion (NMP), which aims to resume metabolism, requiring a more complex perfusion solution that includes an oxygen carrier and nutrients. Whereas NMP is a more complex method, metabolic parameters can be combined with indicators of graft injury to reflect the viability of the donor graft, giving the transplant team an indication whether the organ is suitable for transplantation.

Several research groups have proposed viability criteria to evaluate livers during NMP. Our group has suggested that 2.5 hours of NMP is sufficient to determine whether a liver is potentially transplantable (12). Livers that produced ≥10 grams bile within 2.5 hours and ≥4 grams in the preceding hour were associated with lower levels of transaminases,

while glucose and lactate levels normalized. As a result, bile production was proposed as a viability criterion to identify potentially transplantable ECD livers (12). Watson et al. were the first to report on end-ischemic NMP of suboptimal human livers. Poor prognostic factors were an inability to maintain a normal pH, slow lactate fall and an increase in alanine aminotransferase (ALT) in the perfusion fluid (13). This group also indicated that if bile produced during NMP does not have a pH > 7.45 there is a high risk of post-transplant cholangiopathy (14). The accuracy of bile pH, bicarbonate and glucose as biomarkers of severe bile duct injury was subsequently demonstrated by our group (15,16). Mergental et al. proposed the following criteria after their first series of viability testing during NMP; perfusate lactate <2.5 mmol/L and sufficient bile production in combination with one of the following criteria; pH of perfusate >7.3, stable arterial and portal flow and homogeneous graft perfusion with soft consistency of the parenchyma (17). The criteria for the VITTAL Trial – a machine perfusion study using discarded livers in the UK - are similar, but do not include bile production (18). The viability criteria of the current protocol are based on abovementioned studies and our previous experience.

Perfusion solution with haemoglobin-based oxygen carrier

While sufficient amounts of oxygen can be dissolved in a perfusion solution at temperatures below 20°C, an oxygen carrier is necessary at higher temperatures. Red blood cell (RBC)-based perfusion solutions are therefore mostly used for NMP. RBC are, however, relatively scarce and cannot be used at hypothermic temperatures due to increased stiffness of the erythrocyte lipid membranes which causes hemolysis. Furthermore, immune reactions can be triggered while using RBC (19,20).

Haemoglobin-based oxygen carriers (HBOC) are alternatives for RBC. HBOC-201, a bovine-derived haemoglobin product, has been used in both transfusion and machine perfusion studies (21-23). Recently, HBOC-201 has received FDA-approval for the

application in selected US centers to treat patients with a life-threatening anemia who are not able/willing to receive RBC (21,22). Furthermore, several pre-clinical studies on NMP with an HBOC-based perfusion solution have been performed. Laing et al. have shown that reactive oxygen species production, cell necrosis and apoptosis are not more pronounced in HBOC perfused livers, compared to RBC perfused livers (19). Our group has demonstrated higher bile production and lower (ALT) release during NMP with an HBOC-based perfusion solution, compared with an RBC-based perfusion solution (24). To facilitate machine perfusion at all temperatures and to eliminate the disadvantages of RBC usage, we have developed an HBOC-201-based perfusion solution which will be used for the here described machine perfusion protocol.

End-ischemic *ex situ* machine perfusion protocol

In this protocol we describe a prospective, single-arm study to resuscitate and evaluate initially nationwide declined ECD livers, using a combined protocol of DHOPE, COR, and NMP. Initially nationwide declined ECD livers are statically stored on ice and transported to our centre. DHOPE (8-10 ^{o}C) will then be performed for one hour, to replenish ATP stores and diminish IRI. To facilitate a smooth transition from hypothermia to normothermia, livers will be slowly warmed up to 37°C during an hour, using COR. Subsequently, viability testing will be performed using NMP. If all of the predetermined viability criteria are met, the liver will be transplanted into an eligible recipient. Machine perfusion will be performed using a novel HBOC-201-based perfusion solution for all temperature phases.

METHODS AND ANALYSIS

Study design

This study is a prospective, single-arm, single-centre study. High-risk ECD livers that were declined for liver transplantation by all Dutch liver transplant centres will be accepted for this trial. Typically, these donor livers are at increased risk of developing early graft loss due to PNF, EAD or NAS.

After a donor liver is offered for this study, it will be preserved by SCS and transported to the UMCG. Upon arrival, liver grafts will undergo DHOPE for 1 hour, followed by 1 hour of gradual rewarming until a temperature of 37°C is reached. During 2.5 hours of NMP, a viability assessment will be carried out. The liver will be labeled as transplantable if it meets all predefined viability criteria (Table 1). If a liver meets all viability criteria, the transplant procedure will proceed. If not, the liver will be secondary discarded. **Figure 1** shows a timeline from procurement of the donor liver until transplantation or secondary discard.

Surgical procedure, post-operative care, and follow-up are identical to our routine liver transplantation practice. Patients will be monitored during their hospital stay, and subsequently at routine visits (1 month and 3 months post-transplantation). After three months, graft survival will be evaluated to assess short-term complications; if the 3-months graft-survival is less than 80% the trial will be stopped. A follow-up period of three months after transplantation is chosen, as PNF and EAD typically occur early after transplantation (25).

Study objective and endpoints

The study described in this protocol aims to increase the number of transplantable donor livers, by resuscitating and evaluating initially nationwide declined ECD livers through a combined protocol of DHOPE, COR and NMP, using an HBOC-based perfusion solution.

Primary study endpoint

Graft survival at 3 months after transplantation.

Secondary study endpoints

- Graft and patient survival at 7 days and 1 month.
- Primary non-function (PNF): absence of or minimal function of a liver graft after transplantation requiring re-transplantation or leading to patient death within 7 days after the procedure (26).
- Early allograft dysfunction (EAD), defined as one or more of the following criteria (27):
  - Recipient serum aspartate aminotransferase (AST) and/or alanine aminotransferase (ALT) >2000 IU/mL within the first 7 days after transplantation
  - International normalized ratio (INR) of ≥1.6 on post-operative day 7
  - Recipient serum bilirubin levels of ≥171 μmol/L (10 mg/dL) on post-operative day 7
- Development of clinically evident NAS.
- Biochemical analysis of graft function and IRI determined by post-operative serum levels of ALT, AST, alkaline phosphatase (ALP), gamma-glutamyl

transferase (γGT), INR, lactate dehydrogenase (LDH), creatinine, platelets and total bilirubin at postoperative day 0-7 and 1 and 3 months.

Donor and recipient in- and exclusion criteria

Adult patients (≥18 years old) on the UMCG waiting list for a liver transplantation, who gave informed consent for this study, will be included. In- and exclusion criteria are summarized in **Table 2**.

Study procedures

Preparation of the liver

The donor liver will be procured by one of the national multi organ procurement teams. A standard surgical technique of *in situ* cold flush via the aorta with 4 - 7 L of University of Wisconsin (UW) cold storage solution (0 – 4°C), supplemented with 50.000 IU of heparin, will be used. If possible, the liver will be procured with a segment of 3-5 cm supratruncal aorta left attached to the coeliac trunk. The portal vein and common bile duct will be kept as long as possible. After procurement, the liver will be flushed via the portal vein with at least 1L of UW cold storage solution. The cystic duct will be ligated, and the bile duct will be gently flushed with Belzer UW cold storage solution. The liver will be kept on ice (static cold storage) during transport to the UMCG where cannulation of the supratruncal aorta, portal vein, and bile duct will be performed. Furthermore a small catheter will be inserted into the caval vein to collect venous perfusate samples.

For peer review only - http://bmjopen.bmj.com/site/about/guidelines.xhtml

Preparation of the perfusion solution

The perfusion solution containing HBOC-201 (Table 3) will be prepared under sterile conditions. The perfusion fluid was developed by our research group and has been used successfully in a pre-clinical study (24). For the purpose of this study the perfusion fluid was evaluated and tested for stability and compatibility by the pharmacy of the UMCG. Sterile preparation of the perfusion fluid will be conducted by the pharmacy of the UMCG.

Perfusion device

The Liver Assist (Organ Assist, Groningen, the Netherlands) is a CE marked (European Union Certification of Safety, Health and Environmental Requirements) machine perfusion device for *ex situ* perfusion of donor livers. The Liver Assist enables perfusion of the liver via the portal vein and hepatic artery, using two centrifugal pumps to provide continuous and pulsatile flow, respectively. The system is pressure controlled, which provides auto regulation of the flow through the liver. The temperature can be set from 8 to 37 °C, and the preservation solution can be oxygenated by two hollow fiber membrane oxygenators.

Machine perfusion settings, viability assessment and subsequent transplantation

The Liver Assist will be primed with the HBOC-201-based perfusion solution. The machine perfusion protocol consists of 1h of DHOPE, followed by 1h of COR, and a minimum of 2.5 h of NMP. During DHOPE, the fraction of inspired oxygen (FiO_2) will be set at a 100% and the O_2-flow at 1 L, as described previously (10). During COR and NMP the FiO_2 and O_2-flow will be adjusted according to arterial pO_2 and venous saturation, where pO_2 should be 10.0 – 13.3 kPa and venous saturation 55-75%. During COR, the temperature and arterial and portal pressure will be gradually increased, as depicted in **Figure 2.**

During the first 2.5 h of NMP, viability assessment will be carried out. The liver should match all of the criteria as described in Table 1. These criteria were derived from literature

For peer review only - http://bmjopen.bmj.com/site/about/guidelines.xhtml

and our own experience and reflect both hepatocellular and biliary viability (12,13,15,17,28). If the liver meets the predefined viability criteria, the recipient operation will be started. NMP of the liver will continue until recipient hepatectomy is nearly complete. The liver will then be disconnected from the machine and immediately flushed with 2 L cold Belzer UW cold storage solution to remove the HBOC-201-based perfusion fluid.

Follow-up

After transplantation, patients will be monitored and treated according to standard post-transplant care. After discharge from the hospital, patients will be evaluated in the outpatient clinic up to 3 months. A summary of outcome parameters is shown in **Table 4**.

Statistics

A total of 10 liver transplantations will be included in this study to determine if the proposed protocol can be applied safely. The intervention group will not be compared to a control group, as the livers that will be used are initially nationwide declined livers and it would not be ethical to transplant these livers directly. It is our main concern that the livers are safely transplanted and can match post-operative outcomes of regularly transplanted livers. Graft survival at 3 months after transplantation was chosen as the primary endpoint, as described in the ‘ Methods section’. An acceptable graft survival would be at least >80%, based on data of regular transplantations (29). If the 3-month graft survival rate is <80%, the trial will be stopped. Primary and secondary outcomes are described under ‘Study objective and endpoints’

For peer review only - http://bmjopen.bmj.com/site/about/guidelines.xhtml

Collection, storage and analyses of data and samples

During machine perfusion and liver transplantation

- Perfusion characteristics, such as flow through the portal vein and hepatic artery, pressure settings for the portal vein and hepatic artery, and temperature will be recorded every fifteen minutes.
- Every half hour arterial perfusate, and every hour venous perfusate, will be sampled for point of care analyses to evaluate liver function. Perfusate samples will be stored at -80 °C for later laboratory analyses, such as the assessment of hepatocellular injury markers and function.
- From COR/NMP onwards bile will be collected and stored at -80 °C for later assessment of biliary injury. Furthermore, every half hour bile samples will be collected under mineral oil for point of care analysis to determine cholangiocyte function.
- Biopsies of liver parenchyma and common bile duct will be taken prior to machine perfusion, after machine perfusion and after reperfusion in the recipient. Biopsies will be snap frozen or stored in formalin for paraffin embedding. Hematoxylin and eosin (H&E) staining of paraffin biopsies will be performed to analyze hepatobiliary histological injury.
- Hemodynamic status (mean arterial pressure, heart rate and the use of inotropic medication) of the recipient will be recorded prior to, during and after graft reperfusion.

Other study parameters: Baseline values and parameters at inclusion

- Patient general demographics (age, gender, weight, height).
- Patient medical history, including:

    - Model for end-stage liver disease (MELD) score;
    - Indication for transplantation;
    - Symptoms of decompensated cirrhosis (i.e. ascites; encephalopathy; hepatorenal syndrome)
    - Viral status (i.e. hepatitis A – E; CMV; EBV)
    - Current hospitalization status (home, hospital ward or ICU);
    - Kidney function, as assessed by creatinine, glomerular filtration rate and the need for dialysis;
    - Medication.
- Donor and liver graft characteristics (age, gender, weight before and after machine perfusion, height, cause of death, viral status, liver function, amount of steatosis and donor risk index).
- Reason for initial decline of the liver graft.
- In case of DCD, characteristics such as time interval between withdrawal of life support and circulatory arrest, time interval between circulatory arrest and start cold perfusion *in situ,* donor hepatectomy time.
- Surgical methods and technical difficulties or abnormalities.
- Cold ischemia time, total preservation time (including SCS, DHOPE and NMP) and warm ischemia time during implantation.

For peer review only - http://bmjopen.bmj.com/site/about/guidelines.xhtml

Monitoring and reporting

Serious adverse events (SAE) will be reported to the accredited medical ethical committee through the national web portal *ToetsingOnline* within 15 days after detection.

Patient and public involvement.

Patients and/or public were not involved in the design of the study and the selection of outcome parameters. No patients are involved in the recruitment and/or conduct of the study. Dissemination of the results of the study will be via scientific congresses and meetings, publication(s) in peer-reviewed scientific journal(s), and may include a press release for lay media. Participants in the trial will be informed about the outcomes through their local investigators.

DISCUSSION

ECD livers are increasingly accepted for transplantation, due to the persistent shortage of suitable donor livers, leading to waiting list morbidity and mortality. Although ECD livers are increasingly used, many livers are still declined for transplantation because the estimated risk of early graft loss after transplantation, such as PNF, EAD or biliary complications, is considered too high (1). The current study aims to increase the number of livers suitable for transplantation by resuscitating and evaluating initially nationwide declined ECD livers with a combined machine perfusion protocol, using an HBOC-based perfusion solution.

The risks associated with participation in this study are related to the use of ECD livers. However, current literature and data from our own research group suggest that the proposed protocol will be beneficial for such livers, leading to acceptable outcome after transplantation. DHOPE has been described to resuscitate mitochondria, increase ATP concentration, and reduce IRI of both the hepatocytes and cholangiocytes (8-10). Subsequently, COR prevents (mitochondrial) damage during and after a sudden temperature shift. Lastly, NMP will be used to assess viability (7,11).

The primary endpoint of this study is based on the increased risk of ECD livers to develop PNF, EAD and post-transplant cholangiopathy (1). As these complications mostly occur within 3 months, graft survival at 3 months after transplantation was chosen as the primary outcome (25). If graft survival is >80 %, we will consider the proposed protocol as feasible and safe. A graft survival of <80 % will be considered a cut-off point for this trial. These percentages are based on outcome of regular liver transplantations (29).

One of the down sides of using high-risk ECD liver that were initially nationwide declined is the inability to add a control group. In our opinion it would be unethical to transplant livers with a perceived high risk of complications, without resuscitating and evaluating these livers with machine perfusion prior to transplantation. For studies that include initially declined donor livers it is not unusual to not include a control group, as the

risk of developing severe complications would be too high. The VITTAL Trial has a similar design (18).

To combine the machine perfusion phases, DHOPE, COR and NMP an HBOC-201-based perfusion solution will be used. *In vivo* side effects of first generation HBOCs have been observed in the past. However, HBOC-201 recently received FDA-approval for application in selected centers to treat patients with life-threatening anemia, who are unable/unwilling to receive RBC (21,22). The use of HBOC-201 for machine perfusion is an *ex situ* application and three different groups have reported favorable preclinical results after machine perfusion with an HBOC-201-based perfusion solution (19,24,30). To mitigate the potential side effects of HBOC-201 liver grafts will be flushed thoroughly with regular Belzer static cold storage solution after the machine perfusion procedure.

In summary, with this protocol of sequential DHOPE, COR and NMP we aim to resuscitate and select initially declined suboptimal donor livers for transplantation, in order to increase the number of transplantable livers. The application of the combined machine perfusion protocol will be considered safe and feasible if graft survival at 3 months after transplantation of these initially declined donor livers is similar to that of regularly transplanted donor livers.

ETHICS AND DISSEMINATION

This study is investigator initiated and was approved by the medical ethical committee of Groningen, METc2016.281 in August 2016. Furthermore, approval was given by the Dutch Transplantation Society and the Eurotransplant organization to use initially nationwide declined donor livers for the proposed protocol. The device used for this study is CE marked, and its use was approved by the competent authority as well. As described in the Methods section the perfusion solution will be prepared by our hospital pharmacy according to good manufacturing practice, to ensure sterility and composition of the product. Monitoring of this study will be carried out by the trial coordination center of the UMCG after inclusion of 1, 5- and 10 transplantations to ensure compliance with the protocol and patient safety in this trial.

Patient data will be stored anonymously and can only be decrypted via a secured document. After 15 years, data will be destroyed. All team members involved with this trial will adhere to the code of good research conduct. The results of this study will be submitted to a peer reviewed international medical journal. Data of this study might be used for side studies.

For peer review only - http://bmjopen.bmj.com/site/about/guidelines.xhtml

REFERENCES

(1) Merion RM, Goodrich NP, Feng S. How can we define expanded criteria for liver donors? J Hepatol 2006 Oct;45(4):484-488.

(2) Orman ES, Mayorga ME, Wheeler SB, Townsley RM, Toro-Diaz HH, Hayashi PH, et al. Declining liver graft quality threatens the future of liver transplantation in the United States. Liver Transpl 2015 Aug;21(8):1040-1050.

(3) Orman ES, Barritt AS,4th, Wheeler SB, Hayashi PH. Declining liver utilization for transplantation in the United States and the impact of donation after cardiac death. Liver Transpl 2013 Jan;19(1):59-68.

(4) NTS jaarverslag 2016. 2018; Available at: https://www.transplantatiestichting.nl/sites/default/files/product/downloads/nts_jaarverslag_2016.pdf.

(5) op den Dries S, Karimian N, Sutton ME, Westerkamp AC, Nijsten MW, Gouw AS, et al. Ex vivo normothermic machine perfusion and viability testing of discarded human donor livers. Am J Transplant 2013 May;13(5):1327-1335.

(6) Schlegel A, Graf R, Clavien PA, Dutkowski P. Hypothermic oxygenated perfusion (HOPE) protects from biliary injury in a rodent model of DCD liver transplantation. J Hepatol 2013 Nov;59(5):984-991.

(7) Minor T, Efferz P, Fox M, Wohlschlaeger J, Luer B. Controlled oxygenated rewarming of cold stored liver grafts by thermally graduated machine perfusion prior to reperfusion. Am J Transplant 2013 Jun;13(6):1450-1460.

(8) Karangwa SA, Dutkowski P, Fontes P, Friend PJ, Guarrera JV, Markmann JF, et al. Machine Perfusion of Donor Livers for Transplantation: A Proposal for Standardized Nomenclature and Reporting Guidelines. Am J Transplant 2016 Oct;16(10):2932-2942.

(9) Schlegel A, Rougemont O, Graf R, Clavien PA, Dutkowski P. Protective mechanisms of end-ischemic cold machine perfusion in DCD liver grafts. J Hepatol 2013 Feb;58(2):278-286.

(10) van Rijn R, Karimian N, Matton APM, Burlage LC, Westerkamp AC, van den Berg, A P, et al. Dual hypothermic oxygenated machine perfusion in liver transplants donated after circulatory death. Br J Surg 2017 Apr 10.

(11) Hoyer DP, Mathe Z, Gallinat A, Canbay AC, Treckmann JW, Rauen U, et al. Controlled Oxygenated Rewarming of Cold Stored Livers Prior to Transplantation: First Clinical Application of a New Concept. Transplantation 2016 Jan;100(1):147-152.

(12) Sutton ME, op den Dries S, Karimian N, Weeder PD, de Boer MT, Wiersema-Buist J, et al. Criteria for viability assessment of discarded human donor livers during ex vivo normothermic machine perfusion. PLoS One 2014 Nov 4;9(11):e110642.

(13) Watson CJ, Kosmoliaptsis V, Randle LV, Russell NK, Griffiths WJ, Davies S, et al. Preimplant Normothermic Liver Perfusion of a Suboptimal Liver Donated After Circulatory Death. Am J Transplant 2016 Jan;16(1):353-357.

(14) Watson CJE, Kosmoliaptsis V, Pley C, Randle L, Fear C, Crick K, et al. Observations on the ex situ perfusion of livers for transplantation. Am J Transplant 2018 Feb 8.

(15) Weeder PD, van Rijn R, Porte RJ. Machine perfusion in liver transplantation as a tool to prevent non-anastomotic biliary strictures: Rationale, current evidence and future directions. J Hepatol 2015 Jul;63(1):265-275.

(16) Matton APM, de Vries Y, Burlage LC, van Rijn R, Fujiyoshi M, de Meijer VE, et al. Biliary Bicarbonate, pH and Glucose Are Suitable Biomarkers of Biliary Viability During Ex Situ Normothermic Machine Perfusion of Human Donor Livers. Transplantation 2018 November 01.

For peer review only - http://bmjopen.bmj.com/site/about/guidelines.xhtml

(17) Mergental H, Perera M, Laing RW, Muiesan P, Isaac JR, Smith A, et al. Transplantation of Declined Liver Allografts Following Normothermic Ex-Situ Evaluation. Am J Transplant 2016 May 19.

(18) Laing RW, Mergental H, Yap C, Kirkham A, Whilku M, Barton D, et al. Viability testing and transplantation of marginal livers (VITTAL) using normothermic machine perfusion: study protocol for an open-label, non-randomised, prospective, single-arm trial. BMJ Open 2017 November 28;7(11):017733.

(19) Laing RW, Bhogal RH, Wallace L, Boteon Y, Neil DAH, Smith A, et al. The use of an Acellular Oxygen Carrier in a Human Liver Model of Normothermic Machine Perfusion. Transplantation 2017 May 18.

(20) Buttari B, Profumo E, Rigano R. Crosstalk between red blood cells and the immune system and its impact on atherosclerosis. Biomed Res Int 2015;2015:616834.

(21) Davis JM, El-Haj N, Shah NN, Schwartz G, Block M, Wall J, et al. Use of the blood substitute HBOC-201 in critically ill patients during sickle crisis: a three-case series. Transfusion 2018 Jan;58(1):132-137.

(22) Mer M, Hodgson E, Wallis L, Jacobson B, Levien L, Snyman J, et al. Hemoglobin glutamer-250 (bovine) in South Africa: consensus usage guidelines from clinician experts who have treated patients. Transfusion 2016 October 01;56(10):2631-2636.

(23) Fontes P, Lopez R, van der Plaats A, Vodovotz Y, Minervini M, Scott V, et al. Liver preservation with machine perfusion and a newly developed cell-free oxygen carrier solution under subnormothermic conditions. Am J Transplant 2015 Feb;15(2):381-394.

(24) Matton APM, Burlage LC, van Rijn R, de Vries Y, Karangwa SA, Nijsten MW, et al. Normothermic Machine Perfusion of Donor Livers Without the Need for Human Blood Products. Liver Transpl 2017 Dec 27.

(25) Verhoeven CJ, Farid WR, de Jonge J, Metselaar HJ, Kazemier G, van der Laan, L J. Biomarkers to assess graft quality during conventional and machine preservation in liver transplantation. J Hepatol 2014 Sep;61(3):672-684.

(26) Uemura T, Randall HB, Sanchez EQ, Ikegami T, Narasimhan G, McKenna GJ, et al. Liver retransplantation for primary nonfunction: analysis of a 20-year single-center experience. Liver Transpl 2007 Feb;13(2):227-233.

(27) Olthoff KM, Kulik L, Samstein B, Kaminski M, Abecassis M, Emond J, et al. Validation of a current definition of early allograft dysfunction in liver transplant recipients and analysis of risk factors. Liver Transpl 2010 Aug;16(8):943-949.

(28) Watson CJ, Kosmoliaptsis V, Randle LV, Gimson AE, Brais R, Klinck JR, et al. Normothermic perfusion in the assessment and preservation of declined livers prior to transplantation: hyperoxia and vasoplegia - important lessons from the first 12 cases. Transplantation 2017 Jan 25.

(29) NTS jaarverslag 2015. Available at: . Accessed April, 2017.

(30) Fontes P, Lopez R, van der Plaats A, Vodovotz Y, Minervini M, Scott V, et al. Liver preservation with machine perfusion and a newly developed cell-free oxygen carrier solution under subnormothermic conditions. Am J Transplant 2015 Feb;15(2):381-394.

For peer review only - http://bmjopen.bmj.com/site/about/guidelines.xhtml

LIST OF ABBREVIATONS

ALP	Alkaline phosphatase
ALT	Alanine aminotransferase
AST	Aspartate aminotransferase
ATP	Adenosine 5'-triphosphate
COR	Controlled oxygenated rewarming
DCD	Donation after circulatory death
DHOPE	Dual hypothermic oxygenated perfusion
EAD	Early allograft dysfunction
ECD	Extended criteria donor
HBOC	Hemoglobin-based oxygen carrier
HMP	Hypothermic machine perfusion
INR	International normalized ratio
LDH	Lactate dehydrogenase
NAS	Non-anastomotic biliary strictures
NMP	Normothermic machine perfusion
PNF	Primary non-function
RBC	Red blood cells
SCS	Static cold storage
UMCG	University Medical Center Groningen
UW	University of Wisconsin
γGT	Gamma-glutamyl transferase

AUTHORS CONTRIBUTIONS

The study was initiated and designed by YdV and RJP. All authors contributed to the final study design, which was determined during several meetings. The manuscript was drafted by YdV, TB en RJP. All authors edited the manuscript and approved the final version of the manuscript.

For peer review only - http://bmjopen.bmj.com/site/about/guidelines.xhtml

FUNDING STATEMENT

This work is supported by the Dutch Ministry of Health Welfare and Sport and the de Cock - Hadders Foundation, Groningen. The HBOC-201 is kindly provided by HBO_2 Therapeutics (Souderton, USA).

COMPETING INTEREST STATEMENT

The authors do have no conflict of interest to declare.

For peer review only - http://bmjopen.bmj.com/site/about/guidelines.xhtml

TABLES

Table 1. Viability criteria

- Cumulative bile production of ≥10 mL during first 150 min of NMP and ≥4 mL in the last hour (12).
- Lactate concentration in perfusate is [0.5 – 1.7 mmol/L] within 150 min of NMP (13,17).
- Perfusion fluid pH is [7.35 – 7.45] within 150 min of NMP, without the need for repeated addition of $NaHCO_3$ (13,17).
- Biliary pH of >7.45 within 150 minutes of NMP (15,28).

A liver graft will be deemed transplantable, if all of the criteria were met within 150 min of NMP. As described in the Introduction and in the Methods section, criteria were based on previous studies and own experience, and reflect both hepatocellular and biliary viability.

Table 2. In- and exclusion criteria.

	Inclusion	Exclusion
**Donor**	Body weight ≥40 kg	HIV, hepatitis B or C positive<br>Split or partial liver grafts<br>Domino donor livers<br>Expected CIT ≥10 hours [20]
**Recipient**	≥18 years old<br>Informed consent	HIV positive<br>Mental incapacitation<br>Fulminant liver failure or re-transplantation for PNF<br>Participation in another trial which might influence outcomes of this trial

Abbreviations: CIT, cold ischemia time; HIV, human immunodeficiency virus; PNF, primary non-function.

For peer review only - http://bmjopen.bmj.com/site/about/guidelines.xhtml

Table 3. Composition of the HBOC-201 based perfusion solution.

Component	Manufacturer/Distributor	Volume (mL)
HBOC-201 (Hemopure)	HbO_2 Therapeutics LCC, PA	1250
Gelofusine 4%	B Braun, Melsungen, Germany	300
Albumin 20%	Sanquin® (Dutch blood bank)	250
Total parenteral nutrition (TPN N14G30E)	Hospital pharmacy	20
Addamel (trace elements)	Fresenius Kabi, Netherlands	10
Metronidazol (Flagyl) 5 mg/ml	Baxter BV, Utrecht, Netherlands	44
Sterile water	B Braun, Melsungen, Germany	335
Insulin (NovoRapid) 100 IU/ml	Novo Nordisk BV, Alphen aan den Rijn, Netherlands	1
Cernevit (Multi vitamins)	Baxter BV, Utrecht, Netherlands	2
Heparin (5000 IU/ml)	Leo Pharma, Amsterdam Netherlands	2
Cefazolin 1g /5mL	Baxter BV, Utrecht, Netherlands	2
Taurocholic acid sodium salt 0.1% (1mg/mL)	Hospital pharmacy	7,7
Sodium bicarbonate 8.4%	B Braun, Melsungen, Germany	35
KCl 1mmol/mL	B Braun, Melsungen, Germany	2
Glutathion (Tationil) 600mg/4mL	Teofarma, Pavia, Italy	14
Total volume		2274,7

Table 4. Laboratory investigations during follow-up.

Aspartate aminotransferase
Alanine aminotransferase
Alkaline phosphatase
Gamma-glutamyl transferase
Total bilirubin
Lactate dehydrogenase
Creatinine
Platelets
International normalized ratio

FIGURES LEGENDS

Figure 1. Timeline of a regular liver transplantation vs. the described protocol. The colored bar depicts the machine perfusion protocol. The blue bar represents 1h of DHOPE, the orange bar represents 1h of COR and the red bar represents NMP. If the liver is deemed transplantable within 150 minutes of NMP, NMP will continue (indicated in red). Note that MOD, SCS, transport, and hepatectomy time are approximate. Abbreviations: COR, controlled oxygenated rewarming; DHOPE, dual hypothermic oxygenated perfusion MOD, multi organ donation; NMP, normothermic machine perfusion; OR, operating room; SCS, static cold storage; UMCG, University Medical Center Groningen.

Figure 2. Liver Assist pressure and temperature settings during HMP, COR and NMP. Abbreviations: COR, controlled oxygenated rewarming; DHOPE, dual hypothermic oxygenated perfusion; HA, hepatic artery; NMP, normothermic machine perfusion; PV, portal vein.

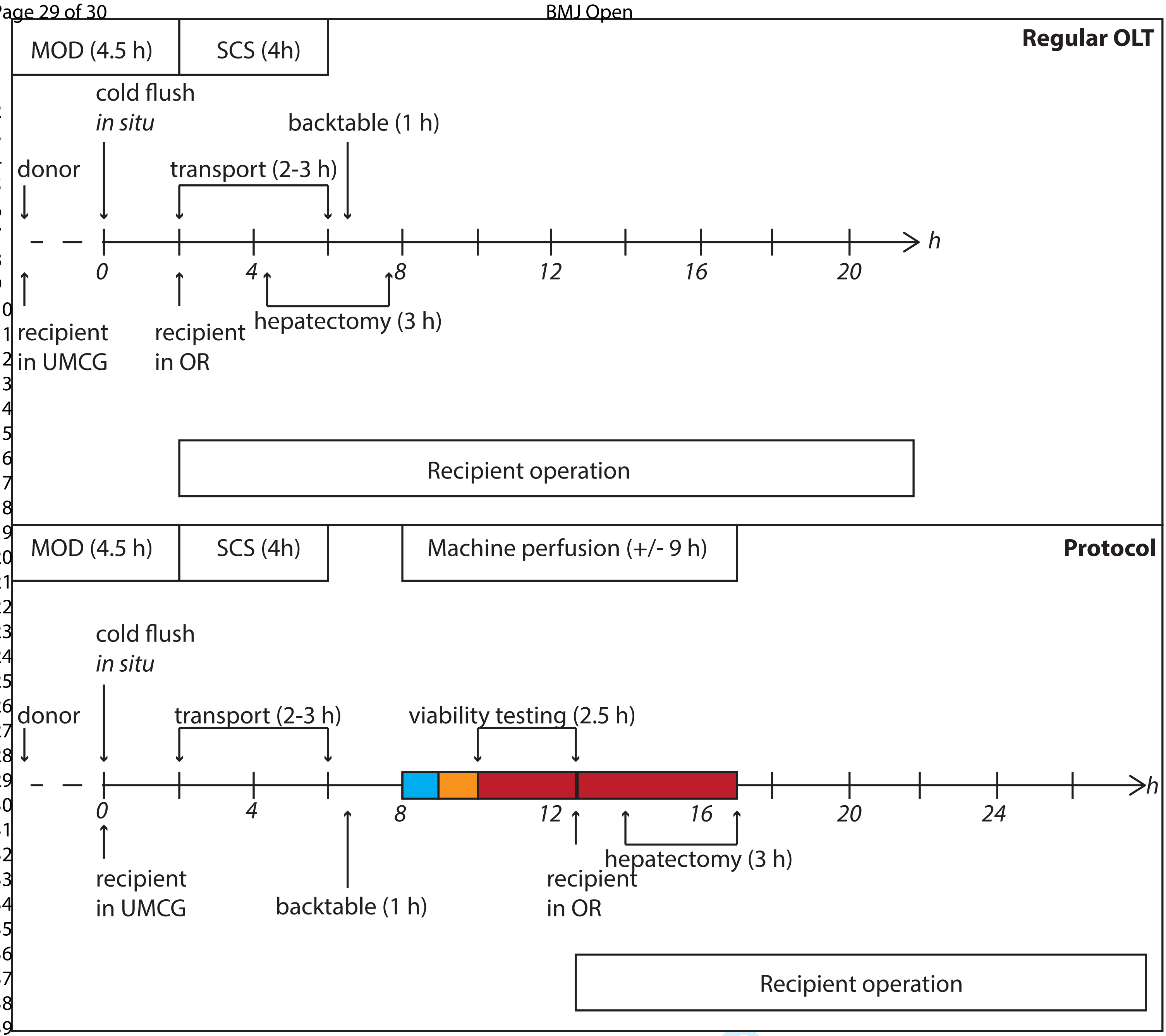
MOD (4.5 h)
SCS (4h)
Regular OLT
cold flush
in situ
backtable (1 h)
donor
transport (2-3 h)
h
0
4
8
12
16
20
hepatectomy (3 h)
recipient
in UMCG
recipient
in OR
Recipient operation
MOD (4.5 h)
SCS (4h)
Machine perfusion (+/- 9 h)
Protocol
cold flush
in situ
donor
transport (2-3 h)
viability testing (2.5 h)
h
0
4
8
12
16
20
24
hepatectomy (3 h)
recipient
in UMCG
backtable (1 h)
recipient
in OR
Recipient operation

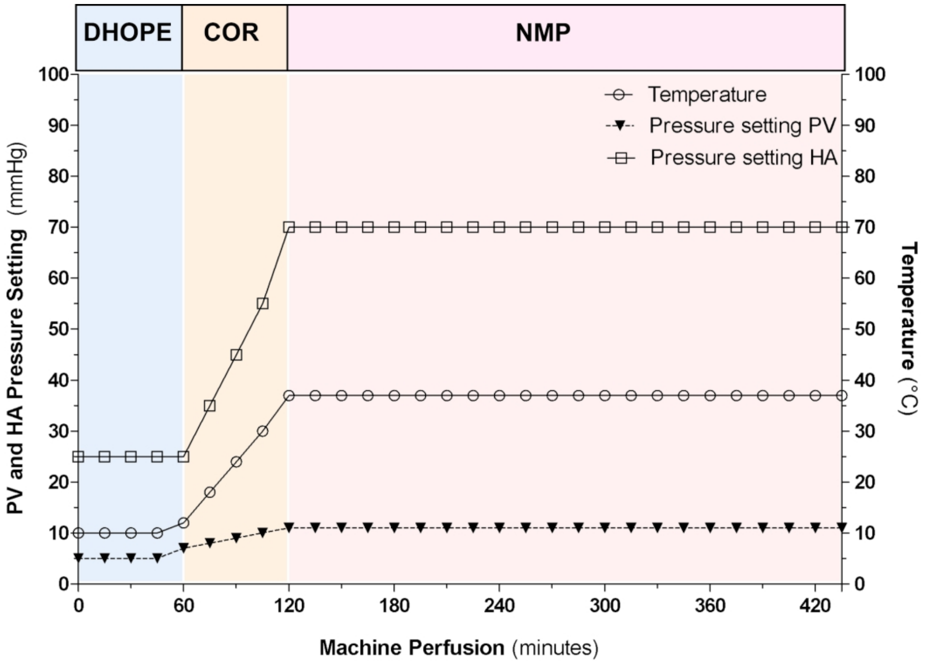
DHOPE
COR
NMP
Temperature
Pressure setting PV
Pressure setting HA
PV and HA Pressure Setting (mmHg)
Temperature (°C)
Machine Perfusion (minutes)
0
60
120
180
240
300
360
420
0
10
20
30
40
50
60
70
80
90
100

Figure 2

BMJ Open

Transplantation of high-risk donor livers after resuscitation and viability assessment using a combined protocol of oxygenated hypothermic, rewarming and normothermic machine perfusion: Study protocol for a prospective, single arm study (DHOPE – COR – NMP Trial)

Journal:	*BMJ Open*
Manuscript ID	bmjopen-2018-028596.R1
Article Type:	Protocol
Date Submitted by the Author:	27-Mar-2019

Complete List of Authors:	de Vries, Yvonne; University Medical Center Groningen, Hepatobiliary surgery and Liver Transplantation<br>Berendsen, Tim ; Hepatobiliary Surgery and Liver Transplantation<br>Fujiyoshi, Masato; Hepatobiliary Surgery and Liver Transplantation<br>van den Berg, Aad; Gasteroenterology and Hepatology<br>Blokzijl, Hans; Universitair Medisch Centrum Groningen, Gastroenterology and Hepatology<br>de Boer, Marieke; Hepatobiliary Surgery and Liver Transplantation<br>van der Heide , Frans; Gasteroenterology and Hepatology<br>de Kleine, Ruben; Hepatobiliary Surgery and Liver Transplantation<br>van Leeuwen, Otto; Hepatobiliary Surgery and Liver Transplantation<br>Matton, Alix; Hepatobiliary Surgery and Liver Transplantation<br>Werner, Maureen; Hepatobiliary Surgery and Liver Transplantation<br>De Meijer, Vincent; Universitair Medisch Centrum Groningen, Surgery<br>Lisman, Ton; Surgical Research Laboratory<br>Porte, Robert; Universitair Medisch Centrum Groningen, Hepatobiliary Surgery and Liver Transplantation

**Primary Subject Heading**:	Surgery
Secondary Subject Heading:	Gastroenterology and hepatology, Surgery
Keywords:	Extended criteria donor liver, Machine perfusion, Liver transplantation, Viability testing, Hemoglobin-based oxygen carrier

SCHOLARONE™
Manuscripts

Transplantation of high-risk donor livers after resuscitation and viability assessment using a combined protocol of oxygenated hypothermic, rewarming and normothermic machine perfusion: Study protocol for a prospective, single arm study (DHOPE – COR – NMP Trial)

Yvonne de Vries[1,2], Tim A. Berendsen[1], Masato Fujiyoshi[1], Aad P. van den Berg[3], Hans Blokzijl[3], Marieke T. de Boer[1], Frans van der Heide[3], Ruben H. J. de Kleine[1], Otto B. van Leeuwen[1,2], Alix P. M. Matton[1,2], Maureen J.M. Werner[1], Ton Lisman[2], Vincent E. de Meijer[1], Robert J. Porte[1]

1) Section Hepatobiliary Surgery and Liver Transplantation, Department of Surgery, University of Groningen, University Medical Center Groningen, Groningen, The Netherlands

2) Surgical Research Laboratory, Department of Surgery, University of Groningen, University Medical Center Groningen, Groningen, The Netherlands

3) Department of Gastroenterology and Hepatology, University of Groningen, University Medical Center Groningen, Groningen, The Netherlands

Corresponding author:

Robert J. Porte, MD, PhD

Section of HPB Surgery and Liver Transplantation

Department of Surgery

University Medical Center Groningen

P.O. Box 30.001

9700 RB Groningen

The Netherlands

Telephone: +31-50-3612896

Fax: +31-50-3614873

E-mail: r.j.porte@umcg.nl

Keywords:

- Extended criteria donor liver
- Machine perfusion
- Haemoglobin-based oxygen carrier
- Liver transplantation
- Viability testing

Word count:

Title: 262 characters

Abstract: 291 words

Main text: 4517 words

For peer review only - http://bmjopen.bmj.com/site/about/guidelines.xhtml

ABSTRACT

Introduction: Extended criteria donor (ECD) livers are increasingly accepted for transplantation in an attempt to reduce the gap between the number of patients on the waiting list and the available number of donor livers. ECD livers, however, carry an increased risk of developing primary non-function (PNF), early allograft dysfunction (EAD) or post-transplant cholangiopathy. Ischemia-reperfusion injury (IRI) plays an important role in the development of these complications. Machine perfusion reduces IRI and allows for reconditioning and subsequent evaluation of liver grafts. Single or dual hypothermic oxygenated machine perfusion (DHOPE) (4 – 12 °C) decreases IRI by resuscitation of mitochondria. Controlled oxygenated rewarming (COR) may further reduce IRI by preventing sudden temperature shifts. Subsequent normothermic machine perfusion (NMP) (37 °C) allows for *ex situ* viability assessment to facilitate the selection of ECD livers with a low risk of PNF, EAD, or post-transplant cholangiopathy.

Methods and analysis: This prospective, single arm study is designed to resuscitate and evaluate initially nationwide declined ECD livers. End-ischemic DHOPE will be performed for initial mitochondrial and graft resuscitation, followed by COR of the donor liver to a normothermic temperature. Subsequently, NMP will be continued to assess viability of the liver. Transplantation into eligible recipients will proceed if all predetermined viability criteria are met within the first 150 min of NMP. To facilitate machine perfusion at different temperatures a perfusion solution containing a haemoglobin-based oxygen carrier (HBOC)-based will be used. With this protocol we aim to transplant extra livers. The primary endpoint is graft survival at 3 months after transplantation.

Ethics and dissemination: This protocol was approved by the medical ethical committee of Groningen, METc2016.281 in August 2016 and registered in the Dutch Trial Register: www.trialregister.nl (NTR5972). Inclusion has been started mid-2017 and is expected to be completed in 2019.

For peer review only - http://bmjopen.bmj.com/site/about/guidelines.xhtml

ARTICLE SUMMARY

Strengths and limitations of this study

- This protocol combines the benefits (resuscitation and *ex situ* viability testing) of multiple machine perfusion modalities (DHOPE, COR and NMP);
- The rewarming phase (COR) links DHOPE and NMP and helps to avoid sudden temperature shifts that may cause additional injury to an already compromised donor organ;
- A newly developed perfusion fluid containing a haemoglobin-based oxygen carrier allows for perfusion at different temperatures, yet is not officially registered in the Netherlands;
- Initially nationwide declined high-risk donor livers, which carry an increased risk of early graft failure due to PNF, EAD or post-transplant cholangiopathy, will be accepted for this machine perfusion protocol.

INTRODUCTION

Increased use of ECD livers

Driven by the donor organ shortage, extended criteria donor (ECD) or suboptimal quality livers are increasingly used for transplantation. These livers would have been refused for transplantation in the past, because of donor characteristics (advanced age, obesity, alcohol abuse, long ICU stay with use of vasopressors) or graft pathology (steatosis, donation after circulatory death {DCD}, elevated liver enzymes, history of liver trauma), but are nowadays accepted due to the shortage of suitable donor livers. ECD livers carry an increased risk of developing primary non-function (PNF), early allograft dysfunction (EAD), or post-transplant cholangiopathy (1). These complications are difficult to predict, causing potentially transplantable ECD livers to be declined. It has been predicted that in the USA the donor liver discard rate will exceed 50% in the next two decades (2,3). In 2016, in the Netherlands 235 donation procedures were effectuated, yet 75 liver grafts were not used, equaling a discard rate of 32% (4).

Machine perfusion as a tool to resuscitate ECD livers

In previous animal and pre-clinical human liver studies, machine perfusion has been applied as a tool to improve and evaluate donor livers prior to transplantation (5-7). Many machine perfusion protocols exist, with varying temperature, perfusion solution, and other characteristics. Oxygenated hypothermic machine perfusion (HMP), which is performed at 4 – 12 °C, aims to resuscitate the mitochondria and sustain adenosine 5'-triphosphate (ATP) production through the supply of oxygen, while the cold keeps metabolic rates suppressed (8-10). Schlegel et al. have shown that 2 hours of oxygenated HMP is sufficient to replenish cellular energy stores (9). A pilot study by our research group, which included 30 DCD livers, has shown a 100% graft survival in the dual hypothermic oxygenated perfusion (DHOPE)

group (n=10), compared to 70% in the static cold storage (SCS) group (n=20). Furthermore, the incidence of non-anastomotic strictures (NAS) of the biliary tree was 10% in the DHOPE group, compared to 35% in the SCS group (10). Based on these favorable results, our centre initiated a multicentre randomized controlled trial on DHOPE (ClinicalTrial.gov identifier NCT02584283).

Several research groups have investigated the use of mid/subnormothermic machine perfusion or controlled oxygenated rewarming (COR), performed at 12 – 34 °C (8). COR may function as a bridge between hypo- and normothermic machine perfusion, omitting a sudden temperature shift, but may also have an IRI reducing effect (7). An abrupt temperature shift contributes to mitochondrial dysfunction, and is more pronounced during reperfusion after HMP, than after COR (7,11). COR has also been described to increase cellular energy content in a clinical study. Furthermore, in a clinical trial performed by the group in Essen, ECD graft survival was a 100% at 6 months after transplantation in the COR group, whereas this was 85% in the SCS group (7,11).

Machine perfusion as a tool to evaluate ECD livers

A more accurate determination of organ viability can be provided by normothermic machine perfusion (NMP), which aims to resume metabolism, requiring a more complex perfusion solution that includes an oxygen carrier and nutrients. Whereas NMP is a more complex method, metabolic parameters can be combined with indicators of graft injury to reflect the viability of the donor graft, giving the transplant team an indication whether the organ is suitable for transplantation.

Several research groups have proposed viability criteria to evaluate livers during NMP. Our group has suggested that 2.5 hours of NMP is sufficient to determine whether a liver is potentially transplantable (12). Livers that produced ≥10 grams bile within 2.5 hours and ≥4 grams in the preceding hour were associated with lower levels of transaminases,

while glucose and lactate levels normalized. As a result, bile production was proposed as a viability criterion to identify potentially transplantable ECD livers (12). Watson et al. were the first to report on end-ischemic NMP of suboptimal human livers. Poor prognostic factors were an inability to maintain a normal pH, slow lactate fall and an increase in alanine aminotransferase (ALT) in the perfusion fluid (13). This group also indicated that if bile produced during NMP does not have a pH > 7.45 there is a high risk of post-transplant cholangiopathy (14). The accuracy of bile pH, bicarbonate and glucose as biomarkers of severe bile duct injury was subsequently demonstrated by our group (15,16). Mergental et al. proposed the following criteria after their first series of viability testing during NMP; perfusate lactate <2.5 mmol/L and sufficient bile production in combination with one of the following criteria; pH of perfusate >7.3, stable arterial and portal flow and homogeneous graft perfusion with soft consistency of the parenchyma (17). The criteria for the VITTAL Trial – a machine perfusion study using discarded livers in the UK - are similar, but do not include a minimal amount of bile production (18). The viability criteria of the current protocol are based on abovementioned studies and our previous experience.

Perfusion solution with haemoglobin-based oxygen carrier

While sufficient amounts of oxygen can be dissolved in a perfusion solution at temperatures below 20°C, an oxygen carrier is necessary at higher temperatures. Red blood cell (RBC)-based perfusion solutions are therefore mostly used for NMP. RBC are, however, relatively scarce and cannot be used at hypothermic temperatures due to increased stiffness of the erythrocyte lipid membranes which causes hemolysis. Furthermore, immune reactions can be triggered while using RBC (19,20).

Haemoglobin-based oxygen carriers (HBOC) are alternatives for RBC. HBOC-201, a bovine-derived haemoglobin product, has been used in both transfusion and machine perfusion studies (21-23). Recently, HBOC-201 has received FDA-approval for the

For peer review only - http://bmjopen.bmj.com/site/about/guidelines.xhtml

application in selected US centers to treat patients with a life-threatening anemia who are not able/willing to receive RBC (21,22). Furthermore, several pre-clinical studies on NMP with an HBOC-based perfusion solution have been performed. Laing et al. have shown that reactive oxygen species production, cell necrosis and apoptosis are not more pronounced in HBOC perfused livers, compared to RBC perfused livers (19). Our group has demonstrated higher bile production and lower (ALT) release during NMP with an HBOC-based perfusion solution, compared with an RBC-based perfusion solution (24). To facilitate machine perfusion at all temperatures and to eliminate the disadvantages of RBC usage, we have developed an HBOC-201-based perfusion solution which will be used for the here described machine perfusion protocol.

End-ischemic *ex situ* machine perfusion protocol

In this protocol we describe a prospective, single-arm study to resuscitate and evaluate initially nationwide declined ECD livers, using a combined protocol of DHOPE, COR, and NMP. Initially nationwide declined ECD livers are statically stored on ice and transported to our centre. DHOPE (8-10 ^{o}C) will then be performed for one hour, to replenish ATP stores and diminish IRI. To facilitate a smooth transition from hypothermia to normothermia, livers will be slowly warmed up to 37°C during an hour, using COR. Subsequently, viability testing will be performed using NMP. If all of the predetermined viability criteria are met, the liver will be transplanted into an eligible recipient. Machine perfusion will be performed using a novel HBOC-201-based perfusion solution for all temperature phases.

METHODS AND ANALYSIS

Study design

This study is a prospective, single-arm, single-centre study. High-risk ECD livers that are declined for liver transplantation by all Dutch liver transplant centres will be accepted for this trial. Typically, these donor livers are at increased risk of developing early graft loss due to PNF, EAD or NAS.

Organs will be allocated in compliance with Eurotransplant (ET) rules, as described below. Organs will be preserved by SCS and transported to the UMCG. Upon arrival, liver grafts will undergo DHOPE for 1 hour, followed by 1 hour of gradual rewarming until a temperature of 37°C is reached. During 2.5 hours of NMP, a viability assessment will be carried out. The liver will be labeled as transplantable if it meets all predefined viability criteria (Table 1). If a liver meets all viability criteria, the transplant procedure will proceed. If not, the liver will be offered back to Eurotransplant for re-allocation via the standard rules, or secondarily discarded if no other centre accepts the liver. **Figure 1** shows a timeline from procurement of the donor liver until transplantation or secondary discard/ secondary offer to Eurotransplant for allocation.

Surgical procedure, post-operative care, and follow-up are identical to our routine liver transplantation practice. Patients will be continuously monitored during their hospital stay, and subsequently at routine visits (1 month and 3 months post-transplantation) (25). After three months, graft survival will be evaluated to assess short-term complications; if the 3-months graft-survival is less than 80% the DHOPE-COR-NMP protocol will be considered unsuccessful. A follow-up period of three months after transplantation is chosen, as PNF and EAD typically occur early after transplantation (26). Although the primary study endpoint graft survival is set at 3 months, all transplant recipients will be followed for at least 12 months to monitor for potential late complications.

For peer review only - http://bmjopen.bmj.com/site/about/guidelines.xhtml

Study objective and endpoints

The study described in this protocol aims to increase the number of transplantable donor livers, by resuscitating and evaluating initially nationwide declined ECD livers through a combined protocol of DHOPE, COR and NMP, using an HBOC-based perfusion solution.

Primary study endpoint

The primary endpoint is graft survival at 3 months after transplantation, where graft survival is defined as the absence of retransplantation or patient death. Both overall graft survival and graft survival censored for patient death (decease of the patient with a functioning graft) will be calculated.

Secondary study endpoints

- Graft- and patient survival at 7 days and 3, 6 and 12 months.
- Primary non-function (PNF): absence of or minimal function of a liver graft after transplantation requiring re-transplantation or leading to patient death within 7 days after the procedure (27).
- Early allograft dysfunction (EAD), defined as one or more of the following criteria (28):
    - Recipient serum aspartate aminotransferase (AST) and/or alanine aminotransferase (ALT) >2000 IU/mL within the first 7 days after transplantation.
    - International normalized ratio (INR) of ≥1.6 on post-operative day 7
    - Recipient serum bilirubin levels of ≥171 μmol/L (10 mg/dL) on post-operative day 7.

For peer review only - http://bmjopen.bmj.com/site/about/guidelines.xhtml

- Development of clinically evident NAS (biliary strictures in presence of symptoms such as elevated cholestatic enzymes, jaundice, cholangitis or pruritus).
- Biochemical analysis of graft function and IRI determined by post-operative serum levels of ALT, AST, alkaline phosphatase (ALP), gamma-glutamyl transferase (γGT), INR, lactate dehydrogenase (LDH), creatinine, platelets and total bilirubin at postoperative day 0-7 and 1 and 3 months.

Donor and recipient in- and exclusion criteria

Adult patients (≥18 years old) on the UMCG waiting list for a liver transplantation, who gave informed consent for this study, will be included. In- and exclusion criteria are summarized in **Table 2**. Eligible patients will be asked for informed consent during the screening process for liver transplantation by a hepatologist or researcher who is well known with the topic and assigned to this task by the delegation log.

Donor risk factors precluding safe transplantation, such as concurrent donor malignancy and a fibrotic/cirrhotic macroscopic appearance of the liver are considered exclusion criteria for the study. Furthermore, an agonal phase of >2 hours in DCD donors is considered an exclusion criteria, as it is national policy for procurement teams to withdraw if circulatory arrest has not occurred within 2 hours after withdrawal of life support. However, formally long donor warm ischemia time is not an exclusion criterion for this study.

Study procedures

Allocation procedure

After the liver of a donor in the Netherlands is declined by all three centres for regular transplantation following static cold storage alone, it will again be offered to our centre for

For peer review only - http://bmjopen.bmj.com/site/about/guidelines.xhtml

inclusion in this protocol. In compliance with ET rules, livers will be allocated only to patients who are identified in the ET match list, based on the ET allocation rules. If a liver is not accepted for a patient who has given informed consent for the DHOPE-COR-NMP trial, it will be allocated to another center in one of the ET member states, according to ET allocation rules. ET and the national competent authority, the Dutch Transplantation Foundation (Nederlandse Transplantatie Stichting) have agreed that this protocol does not interfere with the regular allocation rules within the Netherlands or ET.

Preparation of the liver

The donor liver will be procured by one of the national multi organ procurement teams. A standard surgical technique of *in situ* cold flush via the aorta with 4 - 7 L of University of Wisconsin (UW) cold storage solution (0 – 4°C), supplemented with 50.000 IU of heparin, will be used. If possible, the liver will be procured with a segment of 3-5 cm supratruncal aorta left attached to the coeliac trunk. The portal vein and common bile duct will be kept as long as possible. After procurement, the liver will be flushed via the portal vein with at least 1L of UW cold storage solution. The cystic duct will be ligated, and the bile duct will be gently flushed with Belzer UW cold storage solution. The liver will be kept on ice (static cold storage) during transport to the UMCG where cannulation of the supratruncal aorta, portal vein, and bile duct will be performed. Furthermore a small catheter will be inserted into the caval vein to collect venous perfusate samples.

Preparation of the perfusion solution

The perfusion solution containing HBOC-201 (Table 3) and taurocholic acid for continuous infusion will be prepared under sterile conditions. The perfusion fluid was developed by our research group and has been used successfully in a pre-clinical study and the first clinical patients (24,25). For the purpose of this study the perfusion fluid was evaluated and tested for stability and compatibility by the pharmacy of the UMCG. Sterile preparation of the

For peer review only - http://bmjopen.bmj.com/site/about/guidelines.xhtml

perfusion fluid will be conducted by the pharmacy of the UMCG. Taurocholic acid will be continuously infused into the perfusion solution at a rate of 7.7 mg/h from the start of the NMP phase. Furthermore, sodium-bicarbonate can be added to the perfusion solution to correct a low pH.

Perfusion device

The Liver Assist (Organ Assist, Groningen, the Netherlands) is a CE marked (European Union Certification of Safety, Health and Environmental Requirements) machine perfusion device for *ex situ* perfusion of donor livers. The Liver Assist enables perfusion of the liver via the portal vein and hepatic artery, using two centrifugal pumps to provide continuous and pulsatile flow, respectively. The system is pressure controlled, which provides auto regulation of the flow through the liver. The temperature can be set from 8 to 37 °C, and the preservation solution can be oxygenated by two hollow fiber membrane oxygenators.

Machine perfusion settings, viability assessment and subsequent transplantation

The Liver Assist will be primed with the HBOC-201-based perfusion solution. The machine perfusion protocol consists of 1h of DHOPE, followed by 1h of COR, and a minimum of 2.5 h of NMP. During DHOPE, the fraction of inspired oxygen (FiO_2) will be set at a 100% and the O_2-flow at 1 L, as described previously (10). During COR and NMP the FiO_2 and O_2-flow will be adjusted according to arterial pO_2 and venous saturation at the level of the suprahepatic inferior vena cava, where arterial pO_2 should be 10.0 – 13.3 kPa and venous saturation 55-75%. During COR, the temperature and arterial and portal pressure will be gradually increased, as depicted in **Figure 2.**

During the first 2.5 h of NMP, viability assessment will be carried out. The liver should match all of the criteria as described in Table 1. These criteria were derived from literature

For peer review only - http://bmjopen.bmj.com/site/about/guidelines.xhtml

and our own experience and reflect both hepatocellular and biliary viability (12,13,15,17,29). If the liver meets the predefined viability criteria, the recipient operation will be started. NMP of the liver will continue until recipient hepatectomy is nearly complete. The liver will then be disconnected from the machine and immediately flushed with 2 L cold Belzer UW cold storage solution to remove the HBOC-201-based perfusion fluid. If the liver does not meet the predefined viability criteria, the liver will be offered back to Eurotransplant for re-allocation or secondary discard.

Follow-up

After transplantation, patients will be monitored and treated according to standard post-transplant care. After discharge from the hospital, patients will be evaluated in the outpatient clinic up to 3 months. A summary of outcome parameters is shown in **Table 4**.

Statistics

A total of 10 liver transplantations will be included in this study to determine if the proposed protocol can be applied safely. To transplant 10 livers, we expect to assess 20 nationwide declined donor livers, as our preclinical study has indicated a potential recovery rate of at least 50% (30). The intervention group will not be compared to a control group, as the livers that will be used are initially nationwide declined livers and it would not be ethical to transplant these livers directly. It is our main concern that the livers are safely transplanted and can match post-operative outcomes of regularly transplanted livers. Graft survival at 3 months after transplantation was chosen as the primary endpoint, as described in the ' Methods section'. An acceptable graft survival would be at least >80%, based on data of regular transplantations (31). If the 3-month graft survival rate is <80%, the trial will be stopped. Primary and secondary outcomes are described under 'Study objective and

For peer review only - http://bmjopen.bmj.com/site/about/guidelines.xhtml

Collection, storage and analyses of data and samples

During machine perfusion and liver transplantation

- Perfusion characteristics, such as flow through the portal vein and hepatic artery, pressure settings for the portal vein and hepatic artery, resistance in the portal vein and hepatic artery, and temperature will be recorded every fifteen minutes.
- Every half hour arterial perfusate, and every hour venous perfusate, will be sampled for point of care analyses to evaluate liver function. Perfusate samples will be stored at -80 °C for later laboratory analyses, such as the assessment of hepatocellular injury markers and function.
- From COR/NMP onwards bile will be collected and stored at -80 °C for later assessment of biliary injury. Furthermore, every half hour bile samples will be collected under mineral oil for point of care analysis to determine cholangiocyte function.
- Biopsies of liver parenchyma and common bile duct will be taken prior to machine perfusion, after machine perfusion and after reperfusion in the recipient. Biopsies will be snap frozen or stored in formalin for paraffin embedding. Hematoxylin and eosin (H&E) staining of paraffin biopsies will be performed to analyze hepatobiliary histological injury.
- Hemodynamic status (mean arterial pressure, heart rate and the use of inotropic medication) of the recipient will be recorded prior to, during and after graft reperfusion.

Other study parameters: Baseline values and parameters at inclusion

- Patient general demographics (age, gender, weight, height).
- Patient medical history, including:

For peer review only - http://bmjopen.bmj.com/site/about/guidelines.xhtml

- Model for end-stage liver disease (MELD) score;
- Indication for transplantation;
- Symptoms of decompensated cirrhosis (i.e. ascites; encephalopathy; hepatorenal syndrome)
- Viral status (i.e. hepatitis A – E; CMV; EBV)
- Current hospitalization status (home, hospital ward or ICU);
- Kidney function, as assessed by creatinine, glomerular filtration rate and the need for dialysis;
- Medication.

- Donor and liver graft characteristics (age, gender, weight before and after machine perfusion, height, cause of death, viral status, liver function, amount of steatosis and donor risk index).
- Reason for initial decline of the liver graft.
- In case of DCD, characteristics such as time interval between withdrawal of life support and circulatory arrest, time interval between circulatory arrest and start cold perfusion *in situ,* donor hepatectomy time.
- Surgical methods and technical difficulties or abnormalities.
- Cold ischemia time, total preservation time (including SCS, DHOPE and NMP) and warm ischemia time during implantation.

Monitoring and reporting

Serious adverse events (SAE) will be reported to the accredited medical ethical committee through the national web portal *ToetsingOnline* within 15 days after detection. Monitor visits via the Trial Coordination Center (official institute for the monitoring of clinical trials in our centre) will take place after inclusion of 1-, 5-, and 10 patients. During these visits the monitor will examine the line listing of all adverse events.

Patient and public involvement.

Patients and/or public were not involved in the design of the study and the selection of outcome parameters. No patients are involved in the recruitment and/or conduct of the study. Dissemination of the results of the study will be via scientific congresses and meetings, publication(s) in peer-reviewed scientific journal(s), and may include a press release for lay media. Participants in the trial will be informed about the outcomes through their local investigators.

For peer review only - http://bmjopen.bmj.com/site/about/guidelines.xhtml

DISCUSSION

ECD livers are increasingly accepted for transplantation, due to the persistent shortage of suitable donor livers, leading to waiting list morbidity and mortality. Although ECD livers are increasingly used, many livers are still declined for transplantation because the estimated risk of early graft loss after transplantation, such as PNF, EAD or biliary complications, is considered too high (1). The current study aims to increase the number of livers suitable for transplantation by resuscitating and evaluating initially nationwide declined ECD livers with a combined machine perfusion protocol, using an HBOC-based perfusion solution.

The risks associated with participation in this study are related to the use of ECD livers. However, current literature and data from our own research group suggest that the proposed protocol will be beneficial for such livers, leading to acceptable outcome after transplantation. DHOPE has been described to resuscitate mitochondria, increase ATP concentration, and reduce IRI of both the hepatocytes and cholangiocytes (8-10). Subsequently, COR prevents (mitochondrial) damage during and after a sudden temperature shift. Lastly, NMP will be used to assess viability (7,11).

The primary endpoint of this study is based on the increased risk of ECD livers to develop PNF, EAD and post-transplant cholangiopathy (1). As these complications mostly occur within 3 months, graft survival at 3 months after transplantation was chosen as the primary outcome (26). If graft survival is >80 %, we will consider the proposed protocol as feasible and safe. A graft survival of <80 % will be considered a cut-off point for this trial. These percentages are based on outcome of regular liver transplantations (31).

One of the down sides of using high-risk ECD liver that were initially nationwide declined is the inability to add a control group. In our opinion it would be unethical to transplant livers with a perceived high risk of complications, without resuscitating and evaluating these livers with machine perfusion prior to transplantation. For studies that include initially declined donor livers it is not unusual to not include a control group, as the

risk of developing severe complications would be too high. The VITTAL Trial has a similar design (18).

To combine the machine perfusion phases, DHOPE, COR and NMP an HBOC-201-based perfusion solution will be used. *In vivo* side effects of first generation HBOCs have been observed in the past. However, HBOC-201 recently received FDA-approval for application in selected centers to treat patients with life-threatening anemia, who are unable/unwilling to receive RBC (21,22). The use of HBOC-201 for machine perfusion is an *ex situ* application and three different groups have reported favorable preclinical results after machine perfusion with an HBOC-201-based perfusion solution (19,24,32). To mitigate the potential side effects of HBOC-201 liver grafts will be flushed thoroughly with regular Belzer static cold storage solution after the machine perfusion procedure.

In summary, with this protocol of sequential DHOPE, COR and NMP we aim to resuscitate and select initially declined suboptimal donor livers for transplantation, in order to increase the number of transplantable livers. The application of the combined machine perfusion protocol will be considered safe and feasible if graft survival at 3 months after transplantation of these initially declined donor livers is similar to that of regularly transplanted donor livers.

For peer review only - http://bmjopen.bmj.com/site/about/guidelines.xhtml

ETHICS AND DISSEMINATION

This study is investigator initiated and was approved by the medical ethical committee of Groningen, METc2016.281 in August 2016. Furthermore, approval was given by the Dutch Transplantation Society and the Eurotransplant organization to use initially nationwide declined donor livers for the proposed protocol. The device used for this study is CE marked, and its use was approved by the competent authority as well. As described in the Methods section the perfusion solution will be prepared by our hospital pharmacy according to good manufacturing practice, to ensure sterility and composition of the product. Monitoring of this study will be carried out by the trial coordination center of the UMCG after inclusion of 1, 5- and 10 transplantations to ensure compliance with the protocol and patient safety in this trial.

Patient data will coded and can only be decrypted via a secured document. After 15 years, data will be destroyed. All team members involved with this trial will adhere to the code of good research conduct. The results of this study will be submitted to a peer reviewed international medical journal. Data of this study might be used for side studies.

For peer review only - http://bmjopen.bmj.com/site/about/guidelines.xhtml

REFERENCES

(1) Merion RM, Goodrich NP, Feng S. How can we define expanded criteria for liver donors? J Hepatol 2006 Oct;45(4):484-488.

(2) Orman ES, Mayorga ME, Wheeler SB, Townsley RM, Toro-Diaz HH, Hayashi PH, et al. Declining liver graft quality threatens the future of liver transplantation in the United States. Liver Transpl 2015 Aug;21(8):1040-1050.

(3) Orman ES, Barritt AS,4th, Wheeler SB, Hayashi PH. Declining liver utilization for transplantation in the United States and the impact of donation after cardiac death. Liver Transpl 2013 Jan;19(1):59-68.

(4) NTS jaarverslag 2016. 2018; Available at: https://www.transplantatiestichting.nl/sites/default/files/product/downloads/nts_jaarverslag_2016.pdf.

(5) op den Dries S, Karimian N, Sutton ME, Westerkamp AC, Nijsten MW, Gouw AS, et al. Ex vivo normothermic machine perfusion and viability testing of discarded human donor livers. Am J Transplant 2013 May;13(5):1327-1335.

(6) Schlegel A, Graf R, Clavien PA, Dutkowski P. Hypothermic oxygenated perfusion (HOPE) protects from biliary injury in a rodent model of DCD liver transplantation. J Hepatol 2013 Nov;59(5):984-991.

(7) Minor T, Efferz P, Fox M, Wohlschlaeger J, Luer B. Controlled oxygenated rewarming of cold stored liver grafts by thermally graduated machine perfusion prior to reperfusion. Am J Transplant 2013 Jun;13(6):1450-1460.

(8) Karangwa SA, Dutkowski P, Fontes P, Friend PJ, Guarrera JV, Markmann JF, et al. Machine Perfusion of Donor Livers for Transplantation: A Proposal for Standardized Nomenclature and Reporting Guidelines. Am J Transplant 2016 Oct;16(10):2932-2942.

(9) Schlegel A, Rougemont O, Graf R, Clavien PA, Dutkowski P. Protective mechanisms of end-ischemic cold machine perfusion in DCD liver grafts. J Hepatol 2013 Feb;58(2):278-286.

(10) van Rijn R, Karimian N, Matton APM, Burlage LC, Westerkamp AC, van den Berg, A P, et al. Dual hypothermic oxygenated machine perfusion in liver transplants donated after circulatory death. Br J Surg 2017 Jun;104(7):907-917

(11) Hoyer DP, Mathe Z, Gallinat A, Canbay AC, Treckmann JW, Rauen U, et al. Controlled Oxygenated Rewarming of Cold Stored Livers Prior to Transplantation: First Clinical Application of a New Concept. Transplantation 2016 Jan;100(1):147-152.

(12) Sutton ME, op den Dries S, Karimian N, Weeder PD, de Boer MT, Wiersema-Buist J, et al. Criteria for viability assessment of discarded human donor livers during ex vivo normothermic machine perfusion. PLoS One 2014 Nov 4;9(11):e110642.

(13) Watson CJ, Kosmoliaptsis V, Randle LV, Russell NK, Griffiths WJ, Davies S, et al. Preimplant Normothermic Liver Perfusion of a Suboptimal Liver Donated After Circulatory Death. Am J Transplant 2016 Jan;16(1):353-357.

(14) Watson CJE, Kosmoliaptsis V, Pley C, Randle L, Fear C, Crick K, et al. Observations on the ex situ perfusion of livers for transplantation. Am J Transplant 2018 Aug;18(8):2005-2020

(15) Weeder PD, van Rijn R, Porte RJ. Machine perfusion in liver transplantation as a tool to prevent non-anastomotic biliary strictures: Rationale, current evidence and future directions. J Hepatol 2015 Jul;63(1):265-275.

(16) Matton APM, de Vries Y, Burlage LC, van Rijn R, Fujiyoshi M, de Meijer VE, et al. Biliary Bicarbonate, pH and Glucose Are Suitable Biomarkers of Biliary Viability During Ex Situ Normothermic Machine Perfusion of Human Donor Livers. Transplantation 2018 November 01. doi: 10.1097/TP.0000000000002500.

For peer review only - http://bmjopen.bmj.com/site/about/guidelines.xhtml

(17) Mergental H, Perera M, Laing RW, Muiesan P, Isaac JR, Smith A, et al. Transplantation of Declined Liver Allografts Following Normothermic Ex-Situ Evaluation. Am J Transplant 2016 Nov;16(11):3235-3245.

(18) Laing RW, Mergental H, Yap C, Kirkham A, Whilku M, Barton D, et al. Viability testing and transplantation of marginal livers (VITTAL) using normothermic machine perfusion: study protocol for an open-label, non-randomised, prospective, single-arm trial. BMJ Open 2017 November 28;7(11):017733.

(19) Laing RW, Bhogal RH, Wallace L, Boteon Y, Neil DAH, Smith A, et al. The use of an Acellular Oxygen Carrier in a Human Liver Model of Normothermic Machine Perfusion. Transplantation 2017 Nov;101(11):2746-2756.

(20) Buttari B, Profumo E, Rigano R. Crosstalk between red blood cells and the immune system and its impact on atherosclerosis. Biomed Res Int 2015;2015:616834.

(21) Davis JM, El-Haj N, Shah NN, Schwartz G, Block M, Wall J, et al. Use of the blood substitute HBOC-201 in critically ill patients during sickle crisis: a three-case series. Transfusion 2018 Jan;58(1):132-137.

(22) Mer M, Hodgson E, Wallis L, Jacobson B, Levien L, Snyman J, et al. Hemoglobin glutamer-250 (bovine) in South Africa: consensus usage guidelines from clinician experts who have treated patients. Transfusion 2016 October 01;56(10):2631-2636.

(23) Fontes P, Lopez R, van der Plaats A, Vodovotz Y, Minervini M, Scott V, et al. Liver preservation with machine perfusion and a newly developed cell-free oxygen carrier solution under subnormothermic conditions. Am J Transplant 2015 Feb;15(2):381-394.

(24) Matton APM, Burlage LC, van Rijn R, de Vries Y, Karangwa SA, Nijsten MW, et al. Normothermic Machine Perfusion of Donor Livers Without the Need for Human Blood Products. Liver Transpl 2018 Apr;24(4):528-538

(25) de Vries Y, Matton APM, Nijsten MWN, Werner MJM, van den Berg, A P, de Boer MT, et al. Pretransplant sequential hypo- and normothermic machine perfusion of suboptimal livers donated after circulatory death using a hemoglobin-based oxygen carrier perfusion solution. Am J Transplant 2018 December 26. doi: 10.1111/ajt.15228.

(26) Verhoeven CJ, Farid WR, de Jonge J, Metselaar HJ, Kazemier G, van der Laan, L J. Biomarkers to assess graft quality during conventional and machine preservation in liver transplantation. J Hepatol 2014 Sep;61(3):672-684.

(27) Uemura T, Randall HB, Sanchez EQ, Ikegami T, Narasimhan G, McKenna GJ, et al. Liver retransplantation for primary nonfunction: analysis of a 20-year single-center experience. Liver Transpl 2007 Feb;13(2):227-233.

(28) Olthoff KM, Kulik L, Samstein B, Kaminski M, Abecassis M, Emond J, et al. Validation of a current definition of early allograft dysfunction in liver transplant recipients and analysis of risk factors. Liver Transpl 2010 Aug;16(8):943-949.

(29) Watson CJ, Kosmoliaptsis V, Randle LV, Gimson AE, Brais R, Klinck JR, et al. Normothermic perfusion in the assessment and preservation of declined livers prior to transplantation: hyperoxia and vasoplegia - important lessons from the first 12 cases. Transplantation 2017 Jan 25.

(30) Westerkamp AC, Karimian N, Matton AP, Mahboub P, van Rijn R, Wiersema-Buist J, et al. Oxygenated Hypothermic Machine Perfusion After Static Cold Storage Improves Hepatobiliary Function of Extended Criteria Donor Livers. Transplantation 2016 Apr;100(4):825-835.

(31) NTS jaarverslag 2015. Available at: http://www.transplantatiestichting.nl/sites/default/files/product/downloads/nts_jaarverslag_2015.pdf. Accessed April, 2017.

For peer review only - http://bmjopen.bmj.com/site/about/guidelines.xhtml

(32) Fontes P, Lopez R, van der Plaats A, Vodovotz Y, Minervini M, Scott V, et al. Liver preservation with machine perfusion and a newly developed cell-free oxygen carrier solution under subnormothermic conditions. Am J Transplant 2015 Feb;15(2):381-394.

LIST OF ABBREVIATONS

ALP	Alkaline phosphatase
ALT	Alanine aminotransferase
AST	Aspartate aminotransferase
ATP	Adenosine 5'-triphosphate
COR	Controlled oxygenated rewarming
DCD	Donation after circulatory death
DHOPE	Dual hypothermic oxygenated perfusion
EAD	Early allograft dysfunction
ECD	Extended criteria donor
ET	Eurotransplant
HBOC	Haemoglobin-based oxygen carrier
HMP	Hypothermic machine perfusion
INR	International normalized ratio
LDH	Lactate dehydrogenase
NAS	Non-anastomotic biliary strictures
NMP	Normothermic machine perfusion
PNF	Primary non-function
RBC	Red blood cells
SCS	Static cold storage
UMCG	University Medical Center Groningen
UW	University of Wisconsin
γGT	Gamma-glutamyl transferase

For peer review only - http://bmjopen.bmj.com/site/about/guidelines.xhtml

AUTHORS CONTRIBUTIONS

The study was initiated and designed by YdV and RJP. YdV, MF, APvdB, HB, MTdB, FvdH, RHJdK, OBvL, APMM, MJMW, TL, VEdM and RJP contributed to the final study design, which was determined during several meetings. The manuscript was drafted by YdV, TAB en RJP. YdV, TAB, MF, APvdB, HB, MTdB, FvdH, RHJdK, OBvL, APMM, MJMW, TL, VEdM and RJP edited the manuscript and approved the final version of the manuscript.

FUNDING STATEMENT

This work is supported by the Dutch Ministry of Health Welfare and Sport and the de Cock - Hadders Foundation, Groningen. The HBOC-201 is kindly provided by HBO_2 Therapeutics (Souderton, USA).

COMPETING INTEREST STATEMENT

The authors do have no conflict of interest to declare.

TABLES

Table 1. Viability criteria

- Cumulative bile production of ≥10 mL during first 150 min of NMP and ≥4 mL in the last hour (12).
- Lactate concentration in perfusate is [0.5 – 1.7 mmol/L] within 150 min of NMP (13,17).
- Perfusion fluid pH is [7.35 – 7.45] within 150 min of NMP, without the need for repeated addition of $NaHCO_3$ (13,17).
- Biliary pH of >7.45 within 150 minutes of NMP (15,29).

A liver graft will be deemed transplantable, if all of the criteria were met within 150 min of NMP. As described in the Introduction and in the Methods section, criteria were based on previous studies and own experience, and reflect both hepatocellular and biliary viability.

For peer review only - http://bmjopen.bmj.com/site/about/guidelines.xhtml

Table 2. In- and exclusion criteria.

	Inclusion	Exclusion
**Donor**	Body weight ≥40 kg	HIV, hepatitis B or C positive<br>Split or partial liver grafts<br>Domino donor livers<br>Expected CIT ≥10 hours [20]
**Recipient**	≥18 years old<br>Informed consent	HIV positive<br>Mental incapacitation<br>Fulminant liver failure or re-transplantation for PNF<br>Participation in another trial which might influence outcomes of this trial

Abbreviations: CIT, cold ischemia time; HIV, human immunodeficiency virus; PNF, primary non-function.

Table 3. Composition of the HBOC-201 based perfusion solution.

Component	Manufacturer/Distributor	Volume (mL)
HBOC-201 (Hemopure)	HbO_2 Therapeutics LCC, PA	1250
Gelofusine 4%	B Braun, Melsungen, Germany	300
Albumin 20%	Sanquin® (Dutch blood bank)	250
Total parenteral nutrition (TPN N14G30E)	Hospital pharmacy	20
Addamel (trace elements)	Fresenius Kabi, Netherlands	10
Metronidazol (Flagyl) 5 mg/ml	Baxter BV, Utrecht, Netherlands	44
Sterile water	B Braun, Melsungen, Germany	335
Insulin (NovoRapid) 100 IU/ml	Novo Nordisk BV, Alphen aan den Rijn, Netherlands	1
Cernevit (Multi vitamins)	Baxter BV, Utrecht, Netherlands	2
Heparin (5000 IU/ml)	Leo Pharma, Amsterdam Netherlands	2
Cefazolin 1g /5mL	Baxter BV, Utrecht, Netherlands	2
Taurocholic acid sodium salt 0.1% (1mg/mL)	Hospital pharmacy	7,7
Sodium bicarbonate 8.4%	B Braun, Melsungen, Germany	35
KCl 1mmol/mL	B Braun, Melsungen, Germany	2
Glutathion (Tationil) 600mg/4mL	Teofarma, Pavia, Italy	14
Total volume		2274,7

For peer review only - http://bmjopen.bmj.com/site/about/guidelines.xhtml

Table 4. Laboratory investigations during follow-up.

Aspartate aminotransferase
Alanine aminotransferase
Alkaline phosphatase
Gamma-glutamyl transferase
Total bilirubin
Lactate dehydrogenase
Creatinine
Platelets
International normalized ratio

For peer review only - http://bmjopen.bmj.com/site/about/guidelines.xhtml

FIGURES LEGENDS

Figure 1. Timeline of a regular liver transplantation vs. the described protocol. The colored bar depicts the machine perfusion protocol. The light blue bar represents 1h of DHOPE, the light orange bar represents 1h of COR and the pink bar represents NMP. If the liver is deemed transplantable within 150 minutes of NMP, NMP will continue (indicated in pink). Note that SCS, and transport are approximate. Cold ischemia time (CIT) is defined as the time from the start of cold *in situ* flush in the donor until reperfusion in the recipient. In the proposed protocol CIT is defined as the time from the start of cold flush *in situ* until the start of machine perfusion. Abbreviations: SCS, static cold storage.

Figure 2. Liver Assist pressure and temperature settings during DHOPE, COR and NMP. Abbreviations: COR, controlled oxygenated rewarming; DHOPE, dual hypothermic oxygenated perfusion; HA, hepatic artery; NMP, normothermic machine perfusion; PV, portal vein.

For peer review only - http://bmjopen.bmj.com/site/about/guidelines.xhtml

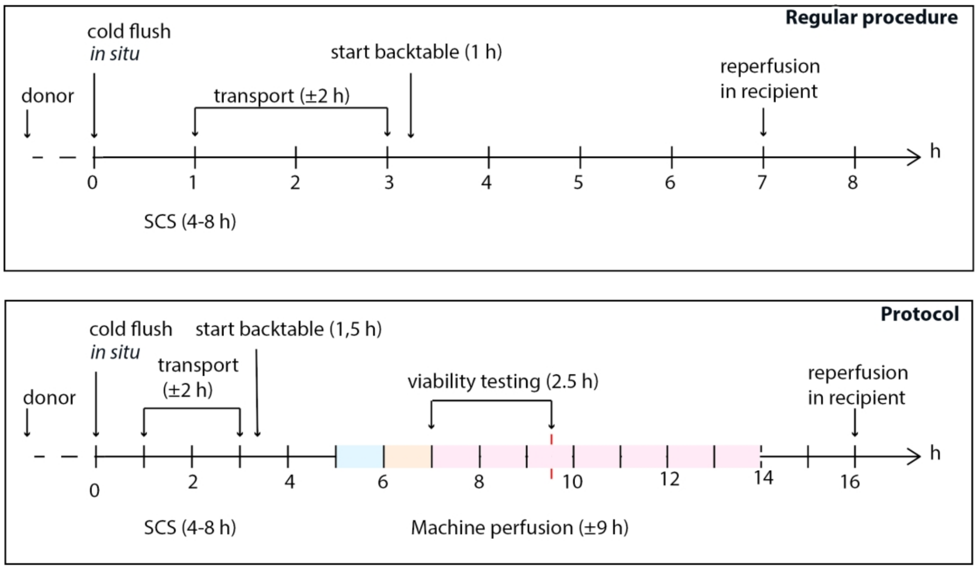
Regular procedure
cold flush
in situ
start backtable (1 h)
reperfusion
in recipient
donor
transport (±2 h)
h
0
1
2
3
4
5
6
7
8
SCS (4-8 h)
Protocol
cold flush
in situ
start backtable (1,5 h)
transport
(±2 h)
viability testing (2.5 h)
reperfusion
in recipient
donor
h
0
2
4
6
8
10
12
14
16
SCS (4-8 h)
Machine perfusion (±9 h)

Figure 1

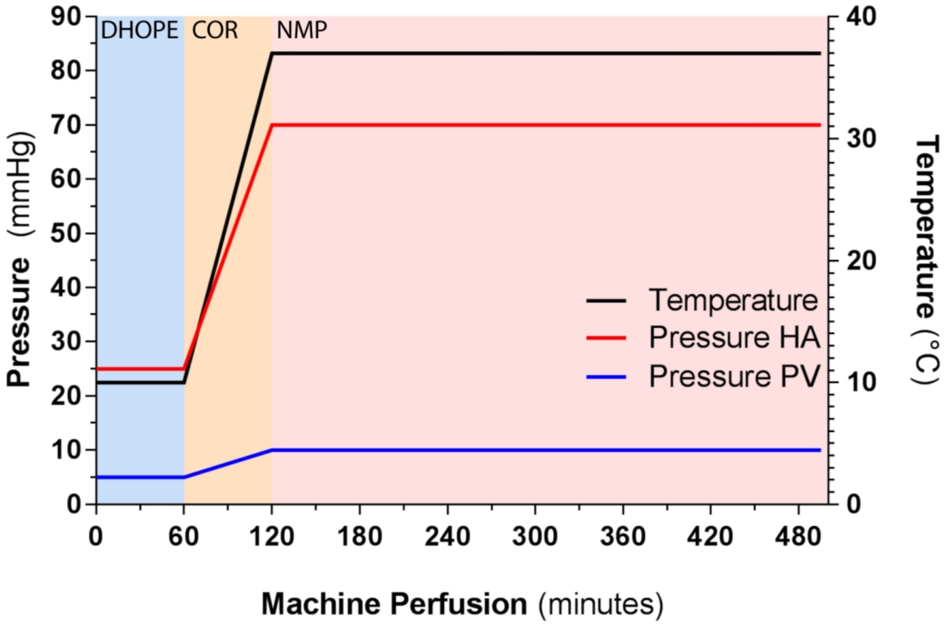
DHOPE
COR
NMP
Pressure (mmHg)
Temperature (°C)
Temperature
Pressure HA
Pressure PV
Machine Perfusion (minutes)
90
80
70
60
50
40
30
20
10
0
40
30
20
10
0
0
60
120
180
240
300
360
420
480

Figure 2

SPIRIT

STANDARD PROTOCOL ITEMS: RECOMMENDATIONS FOR INTERVENTIONAL TRIALS

SPIRIT 2013 Checklist: Recommended items to address in a clinical trial protocol and related documents*

Section/item	Item No	Description	Notes
**Administrative information**			
Title	1	Descriptive title identifying the study design, population, interventions, and, if applicable, trial acronym	✓
Trial registration	2a	Trial identifier and registry name. If not yet registered, name of intended registry	✓ page6
	2b	All items from the World Health Organization Trial Registration Data Set	NA
Protocol version	3	Date and version identifier	
Funding	4	Sources and types of financial, material, and other support	✓ page 25
Roles and responsibilities	5a	Names, affiliations, and roles of protocol contributors	✓ page 1 + 23
	5b	Name and contact information for the trial sponsor	✓ page 1
	5c	Role of study sponsor and funders, if any, in study design; collection, management, analysis, and interpretation of data; writing of the report; and the decision to submit the report for publication, including whether they will have ultimate authority over any of these activities	page 25
	5d	Composition, roles, and responsibilities of the coordinating centre, steering committee, endpoint adjudication committee, data management team, and other individuals or groups overseeing the trial, if applicable (see Item 21a for data monitoring committee)	N.A. for monitoring see page 18
**Introduction**			
Background and rationale	6a	Description of research question and justification for undertaking the trial, including summary of relevant studies (published and unpublished) examining benefits and harms for each intervention	✓ page 5-8
	6b	Explanation for choice of comparators	N.A.
Objectives	7	Specific objectives or hypotheses	✓ page 10
Trial design	8	Description of trial design including type of trial (eg, parallel group, crossover, factorial, single group), allocation ratio, and framework (eg, superiority, equivalence, noninferiority, exploratory)	✓ page 9

For peer review only - http://bmjopen.bmj.com/site/about/guidelines.xhtml

Allocation concealment mechanism	16b	Mechanism of implementing the allocation sequence (eg, central telephone; sequentially numbered, opaque, sealed envelopes), describing any steps to conceal the sequence until interventions are assigned NA
Implementation	16c	Who will generate the allocation sequence, who will enrol participants, and who will assign participants to interventions NA
Blinding (masking)	17a	Who will be blinded after assignment to interventions (eg, trial participants, care providers, outcome assessors, data analysts), and how NA
	17b	If blinded, circumstances under which unblinding is permissible, and procedure for revealing a participant's allocated intervention during the trial NA.

Methods: Data collection, management, and analysis

Data collection methods	18a	Plans for assessment and collection of outcome, baseline, and other trial data, including any related processes to promote data quality (eg, duplicate measurements, training of assessors) and a description of study instruments (eg, questionnaires, laboratory tests) along with their reliability and validity, if known. Reference to where data collection forms can be found, if not in the protocol page 16&17.
	18b	Plans to promote participant retention and complete follow-up, including list of any outcome data to be collected for participants who discontinue or deviate from intervention protocols N.A.
Data management	19	Plans for data entry, coding, security, and storage, including any related processes to promote data quality (eg, double data entry; range checks for data values). Reference to where details of data management procedures can be found, if not in the protocol page21
Statistical methods	20a	Statistical methods for analysing primary and secondary outcomes. Reference to where other details of the statistical analysis plan can be found, if not in the protocol page 14
	20b	Methods for any additional analyses (eg, subgroup and adjusted analyses) N.A.
	20c	Definition of analysis population relating to protocol non-adherence (eg, as randomised analysis), and any statistical methods to handle missing data (eg, multiple imputation) NA.

Methods: Monitoring

Data monitoring	21a	Composition of data monitoring committee (DMC); summary of its role and reporting structure; statement of whether it is independent from the sponsor and competing interests; and reference to where further details about its charter can be found, if not in the protocol. Alternatively, an explanation of why a DMC is not needed N.A. page18

Appendices

Informed consent materials	32	Model consent form and other related documentation given to participants and authorised surrogates seperate from protocol, not included
Biological specimens	33	Plans for collection, laboratory evaluation, and storage of biological specimens for genetic or molecular analysis in the current trial and for future use in ancillary studies, if applicable page 21.

*It is strongly recommended that this checklist be read in conjunction with the SPIRIT 2013 Explanation & Elaboration for important clarification on the items. Amendments to the protocol should be tracked and dated. The SPIRIT checklist is copyrighted by the SPIRIT Group under the Creative Commons "Attribution-NonCommercial-NoDerivs 3.0 Unported" license.

Methods: Participants, interventions, and outcomes

Study setting	9	Description of study settings (eg, community clinic, academic hospital) and list of countries where data will be collected. Reference to where list of study sites can be obtained	NA.
Eligibility criteria	10	Inclusion and exclusion criteria for participants. If applicable, eligibility criteria for study centres and individuals who will perform the interventions (eg, surgeons, psychotherapists)	√ page 11
Interventions	11a	Interventions for each group with sufficient detail to allow replication, including how and when they will be administered	one group √ page 11 - 14
	11b	Criteria for discontinuing or modifying allocated interventions for a given trial participant (eg, drug dose change in response to harms, participant request, or improving/worsening disease)	NA.
	11c	Strategies to improve adherence to intervention protocols, and any procedures for monitoring adherence (eg, drug tablet return, laboratory tests)	N. A.
	11d	Relevant concomitant care and interventions that are permitted or prohibited during the trial	NA.
Outcomes	12	Primary, secondary, and other outcomes, including the specific measurement variable (eg, systolic blood pressure), analysis metric (eg, change from baseline, final value, time to event), method of aggregation (eg, median, proportion), and time point for each outcome. Explanation of the clinical relevance of chosen efficacy and harm outcomes is strongly recommended	√ page 10+11 √ page 15+16 table 4.
Participant timeline	13	Time schedule of enrolment, interventions (including any run-ins and washouts), assessments, and visits for participants. A schematic diagram is highly recommended (see Figure)	√ page 14 see point 12
Sample size	14	Estimated number of participants needed to achieve study objectives and how it was determined, including clinical and statistical assumptions supporting any sample size calculations	√ page 14
Recruitment	15	Strategies for achieving adequate participant enrolment to reach target sample size	√ page 11

Methods: Assignment of interventions (for controlled trials)

Allocation:

Sequence generation	16a	Method of generating the allocation sequence (eg, computer-generated random numbers), and list of any factors for stratification. To reduce predictability of a random sequence, details of any planned restriction (eg, blocking) should be provided in a separate document that is unavailable to those who enrol participants or assign interventions	NA.

For peer review only - http://bmjopen.bmj.com/site/about/guidelines.xhtml

	21b	Description of any interim analyses and stopping guidelines, including who will have access to these interim results and make the final decision to terminate the trial	NA. page 14
Harms	22	Plans for collecting, assessing, reporting, and managing solicited and spontaneously reported adverse events and other unintended effects of trial interventions or trial conduct	page 18
Auditing	23	Frequency and procedures for auditing trial conduct, if any, and whether the process will be independent from investigators and the sponsor	N.A.
**Ethics and dissemination**			
Research ethics approval	24	Plans for seeking research ethics committee/institutional review board (REC/IRB) approval	page 21.
Protocol amendments	25	Plans for communicating important protocol modifications (eg, changes to eligibility criteria, outcomes, analyses) to relevant parties (eg, investigators, REC/IRBs, trial participants, trial registries, journals, regulators)	page 21.
Consent or assent	26a	Who will obtain informed consent or assent from potential trial participants or authorised surrogates, and how (see Item 32)	page 11
	26b	Additional consent provisions for collection and use of participant data and biological specimens in ancillary studies, if applicable	N.A.
Confidentiality	27	How personal information about potential and enrolled participants will be collected, shared, and maintained in order to protect confidentiality before, during, and after the trial	page 21
Declaration of interests	28	Financial and other competing interests for principal investigators for the overall trial and each study site	page 25
Access to data	29	Statement of who will have access to the final trial dataset, and disclosure of contractual agreements that limit such access for investigators	page 21
Ancillary and post-trial care	30	Provisions, if any, for ancillary and post-trial care, and for compensation to those who suffer harm from trial participation	insurance sheet for patients not includen in protocol for publication
Dissemination policy	31a	Plans for investigators and sponsor to communicate trial results to participants, healthcare professionals, the public, and other relevant groups (eg, via publication, reporting in results databases, or other data sharing arrangements), including any publication restrictions	page 21.
	31b	Authorship eligibility guidelines and any intended use of professional writers	N.A.
	31c	Plans, if any, for granting public access to the full protocol, participant-level dataset, and statistical code	N.A.

BMJ Open

Transplantation of high-risk donor livers after resuscitation and viability assessment using a combined protocol of oxygenated hypothermic, rewarming and normothermic machine perfusion: Study protocol for a prospective, single arm study (DHOPE – COR – NMP Trial)

Journal:	*BMJ Open*
Manuscript ID	bmjopen-2018-028596.R2
Article Type:	Protocol
Date Submitted by the Author:	08-Jul-2019
Complete List of Authors:	de Vries, Yvonne; University Medical Center Groningen, Hepatobiliary surgery and Liver Transplantation<br>Berendsen, Tim ; Hepatobiliary Surgery and Liver Transplantation<br>Fujiyoshi, Masato; Hepatobiliary Surgery and Liver Transplantation<br>van den Berg, Aad; Gasteroenterology and Hepatology<br>Blokzijl, Hans; Universitair Medisch Centrum Groningen, Gastroenterology and Hepatology<br>de Boer, Marieke; Hepatobiliary Surgery and Liver Transplantation<br>van der Heide , Frans; Gasteroenterology and Hepatology<br>de Kleine, Ruben; Hepatobiliary Surgery and Liver Transplantation<br>van Leeuwen, Otto; Hepatobiliary Surgery and Liver Transplantation<br>Matton, Alix; Hepatobiliary Surgery and Liver Transplantation<br>Werner, Maureen; Hepatobiliary Surgery and Liver Transplantation<br>Lisman, Ton; Surgical Research Laboratory<br>De Meijer, Vincent; Universitair Medisch Centrum Groningen, Surgery<br>Porte, Robert; Universitair Medisch Centrum Groningen, Hepatobiliary Surgery and Liver Transplantation
**Primary Subject Heading**:	Surgery
Secondary Subject Heading:	Gastroenterology and hepatology, Surgery
Keywords:	Extended criteria donor liver, Machine perfusion, Liver transplantation, Viability testing, Hemoglobin-based oxygen carrier

SCHOLARONE™
Manuscripts

Transplantation of high-risk donor livers after resuscitation and viability assessment using a combined protocol of oxygenated hypothermic, rewarming and normothermic machine perfusion: Study protocol for a prospective, single arm study (DHOPE – COR – NMP Trial)

Yvonne de Vries[1,2], Tim A. Berendsen[1], Masato Fujiyoshi[1], Aad P. van den Berg[3], Hans Blokzijl[3], Marieke T. de Boer[1], Frans van der Heide[3], Ruben H. J. de Kleine[1], Otto B. van Leeuwen[1,2], Alix P. M. Matton[1,2], Maureen J.M. Werner[1], Ton Lisman[2], Vincent E. de Meijer[1], Robert J. Porte[1]

1) Section Hepatobiliary Surgery and Liver Transplantation, Department of Surgery, University of Groningen, University Medical Center Groningen, Groningen, The Netherlands

2) Surgical Research Laboratory, Department of Surgery, University of Groningen, University Medical Center Groningen, Groningen, The Netherlands

3) Department of Gastroenterology and Hepatology, University of Groningen, University Medical Center Groningen, Groningen, The Netherlands

Corresponding author:

Robert J. Porte, MD, PhD

Section of HPB Surgery and Liver Transplantation

Department of Surgery

University Medical Center Groningen

P.O. Box 30.001

9700 RB Groningen

The Netherlands

Telephone: +31-50-3612896

Fax: +31-50-3614873

E-mail: r.j.porte@umcg.nl

Keywords:

- Extended criteria donor liver
- Machine perfusion
- Haemoglobin-based oxygen carrier
- Liver transplantation
- Viability testing

Word count:

Title: 262 characters

Abstract: 291 words

Main text: 4570 words

For peer review only - http://bmjopen.bmj.com/site/about/guidelines.xhtml

ABSTRACT

Introduction: Extended criteria donor (ECD) livers are increasingly accepted for transplantation in an attempt to reduce the gap between the number of patients on the waiting list and the available number of donor livers. ECD livers, however, carry an increased risk of developing primary non-function (PNF), early allograft dysfunction (EAD) or post-transplant cholangiopathy. Ischemia-reperfusion injury (IRI) plays an important role in the development of these complications. Machine perfusion reduces IRI and allows for reconditioning and subsequent evaluation of liver grafts. Single or dual hypothermic oxygenated machine perfusion (DHOPE) (4 – 12 °C) decreases IRI by resuscitation of mitochondria. Controlled oxygenated rewarming (COR) may further reduce IRI by preventing sudden temperature shifts. Subsequent normothermic machine perfusion (NMP) (37 °C) allows for *ex situ* viability assessment to facilitate the selection of ECD livers with a low risk of PNF, EAD, or post-transplant cholangiopathy.

Methods and analysis: This prospective, single arm study is designed to resuscitate and evaluate initially nationwide declined ECD livers. End-ischemic DHOPE will be performed for initial mitochondrial and graft resuscitation, followed by COR of the donor liver to a normothermic temperature. Subsequently, NMP will be continued to assess viability of the liver. Transplantation into eligible recipients will proceed if all predetermined viability criteria are met within the first 150 min of NMP. To facilitate machine perfusion at different temperatures a perfusion solution containing a haemoglobin-based oxygen carrier (HBOC)-based will be used. With this protocol we aim to transplant extra livers. The primary endpoint is graft survival at 3 months after transplantation.

Ethics and dissemination: This protocol was approved by the medical ethical committee of Groningen, METc2016.281 in August 2016 and registered in the Dutch Trial Register: www.trialregister.nl (NTR5972). Inclusion has been started mid-2017 and is expected to be completed in 2019.

For peer review only - http://bmjopen.bmj.com/site/about/guidelines.xhtml

ARTICLE SUMMARY

Strengths and limitations of this study

- This protocol combines the benefits (resuscitation and *ex situ* viability testing) of multiple machine perfusion modalities (DHOPE, COR and NMP);
- The rewarming phase (COR) links DHOPE and NMP and helps to avoid sudden temperature shifts that may cause additional injury to an already compromised donor organ;
- A newly developed perfusion fluid containing a haemoglobin-based oxygen carrier allows for perfusion at different temperatures, yet is not officially registered in the Netherlands;
- Initially nationwide declined high-risk donor livers, which carry an increased risk of early graft failure due to PNF, EAD or post-transplant cholangiopathy, will be accepted for this machine perfusion protocol.

INTRODUCTION

Increased use of ECD livers

Driven by the donor organ shortage, extended criteria donor (ECD) or suboptimal quality livers are increasingly used for transplantation. These livers would have been refused for transplantation in the past, because of donor characteristics (advanced age, obesity, alcohol abuse, long ICU stay with use of vasopressors) or graft pathology (steatosis, donation after circulatory death {DCD}, elevated liver enzymes, history of liver trauma), but are nowadays accepted due to the shortage of suitable donor livers. ECD livers carry an increased risk of developing primary non-function (PNF), early allograft dysfunction (EAD), or post-transplant cholangiopathy (1). These complications are difficult to predict, causing potentially transplantable ECD livers to be declined. It has been predicted that in the USA the donor liver discard rate will exceed 50% in the next two decades (2,3). In 2016, in the Netherlands 235 donation procedures were effectuated, yet 75 liver grafts were not used, equaling a discard rate of 32% (4).

Machine perfusion as a tool to resuscitate ECD livers

In previous animal and pre-clinical human liver studies, machine perfusion has been applied as a tool to improve and evaluate donor livers prior to transplantation (5-7). Many machine perfusion protocols exist, with varying temperature, perfusion solution, and other characteristics. Oxygenated hypothermic machine perfusion (HMP), which is performed at 4 – 12 °C, aims to resuscitate the mitochondria and sustain adenosine 5'-triphosphate (ATP) production through the supply of oxygen, while the cold keeps metabolic rates suppressed (8-10). Schlegel et al. have shown that 2 hours of oxygenated HMP is sufficient to replenish cellular energy stores (9). A pilot study by our research group, which included 30 DCD livers, has shown a 100% graft survival in the dual hypothermic oxygenated perfusion (DHOPE)

group (n=10), compared to 70% in the static cold storage (SCS) group (n=20). Furthermore, the incidence of non-anastomotic strictures (NAS) of the biliary tree was 10% in the DHOPE group, compared to 35% in the SCS group (10). Based on these favorable results, our centre initiated a multicentre randomized controlled trial on DHOPE (ClinicalTrial.gov identifier NCT02584283).

Several research groups have investigated the use of mid/subnormothermic machine perfusion or controlled oxygenated rewarming (COR), performed at 12 – 34 °C (8). COR may function as a bridge between hypo- and normothermic machine perfusion, omitting a sudden temperature shift, but may also have an IRI reducing effect (7). An abrupt temperature shift contributes to mitochondrial dysfunction, and is more pronounced during reperfusion after HMP, than after COR (7,11). COR has also been described to increase cellular energy content in a clinical study. Furthermore, in a clinical trial performed by the group in Essen, ECD graft survival was a 100% at 6 months after transplantation in the COR group, whereas this was 85% in the SCS group (7,11).

Machine perfusion as a tool to evaluate ECD livers

A more accurate determination of organ viability can be provided by normothermic machine perfusion (NMP), which aims to resume metabolism, requiring a more complex perfusion solution that includes an oxygen carrier and nutrients. Whereas NMP is a more complex method, metabolic parameters can be combined with indicators of graft injury to reflect the viability of the donor graft, giving the transplant team an indication whether the organ is suitable for transplantation.

Several research groups have proposed viability criteria to evaluate livers during NMP. Our group has suggested that 2.5 hours of NMP is sufficient to determine whether a liver is potentially transplantable (12). Livers that produced ≥10 grams bile within 2.5 hours and ≥4 grams in the preceding hour were associated with lower levels of transaminases,

while glucose and lactate levels normalized. As a result, bile production was proposed as a viability criterion to identify potentially transplantable ECD livers (12). Watson et al. were the first to report on end-ischemic NMP of suboptimal human livers. Poor prognostic factors were an inability to maintain a normal pH, slow lactate fall and an increase in alanine aminotransferase (ALT) in the perfusion fluid (13). This group also indicated that if bile produced during NMP does not have a pH > 7.45 there is a high risk of post-transplant cholangiopathy (14). The accuracy of bile pH, bicarbonate and glucose as biomarkers of severe bile duct injury was subsequently demonstrated by our group (15,16). Mergental et al. proposed the following criteria after their first series of viability testing during NMP; perfusate lactate <2.5 mmol/L and sufficient bile production in combination with one of the following criteria; pH of perfusate >7.3, stable arterial and portal flow and homogeneous graft perfusion with soft consistency of the parenchyma (17). The criteria for the VITTAL Trial – a machine perfusion study using discarded livers in the UK - are similar, but do not include a minimal amount of bile production (18). The viability criteria of the current protocol are based on abovementioned studies and our previous experience.

Perfusion solution with haemoglobin-based oxygen carrier

While sufficient amounts of oxygen can be dissolved in a perfusion solution at temperatures below 20°C, an oxygen carrier is necessary at higher temperatures. Red blood cell (RBC)-based perfusion solutions are therefore mostly used for NMP. RBC are, however, relatively scarce and cannot be used at hypothermic temperatures due to increased stiffness of the erythrocyte lipid membranes which causes hemolysis. Furthermore, immune reactions can be triggered while using RBC (19,20).

Haemoglobin-based oxygen carriers (HBOC) are alternatives for RBC. HBOC-201, a bovine-derived haemoglobin product, has been used in both transfusion and machine perfusion studies (21-23). Recently, HBOC-201 has received FDA-approval for the

application in selected US centers to treat patients with a life-threatening anemia who are not able/willing to receive RBC (21,22). Furthermore, several pre-clinical studies on NMP with an HBOC-based perfusion solution have been performed. Laing et al. have shown that reactive oxygen species production, cell necrosis and apoptosis are not more pronounced in HBOC perfused livers, compared to RBC perfused livers (19). Our group has demonstrated higher bile production and lower (ALT) release during NMP with an HBOC-based perfusion solution, compared with an RBC-based perfusion solution (24). To facilitate machine perfusion at all temperatures and to eliminate the disadvantages of RBC usage, we have developed an HBOC-201-based perfusion solution which will be used for the here described machine perfusion protocol.

End-ischemic *ex situ* machine perfusion protocol

In this protocol we describe a prospective, single-arm study to resuscitate and evaluate initially nationwide declined ECD livers, using a combined protocol of DHOPE, COR, and NMP. Initially nationwide declined ECD livers are statically stored on ice and transported to our centre. DHOPE (8-10 ^{o}C) will then be performed for one hour, to replenish ATP stores and diminish IRI. To facilitate a smooth transition from hypothermia to normothermia, livers will be slowly warmed up to 37°C during an hour, using COR. Subsequently, viability testing will be performed using NMP. If all of the predetermined viability criteria are met, the liver will be transplanted into an eligible recipient. Machine perfusion will be performed using a novel HBOC-201-based perfusion solution for all temperature phases.

METHODS AND ANALYSIS

Study design

This study is a prospective, single-arm, single-centre study. High-risk ECD livers that are declined for liver transplantation by all Dutch liver transplant centres will be accepted for this trial. Typically, these donor livers are at increased risk of developing early graft loss due to PNF, EAD or NAS.

Organs will be allocated in compliance with Eurotransplant (ET) rules, as described below. Organs will be preserved by SCS and transported to the UMCG. Upon arrival, liver grafts will undergo DHOPE for 1 hour, followed by 1 hour of gradual rewarming until a temperature of 37°C is reached. During 2.5 hours of NMP, a viability assessment will be carried out. The liver will be labeled as transplantable if it meets all predefined viability criteria (Table 1). If a liver meets all viability criteria, the transplant procedure will proceed. If not, the liver will be offered back to Eurotransplant for re-allocation via the standard rules, or secondarily discarded if no other centre accepts the liver. **Figure 1** shows a timeline from procurement of the donor liver until transplantation or secondary discard/ secondary offer to Eurotransplant for allocation.

Surgical procedure, post-operative care, and follow-up are identical to our routine liver transplantation practice. Patients will be continuously monitored during their hospital stay, and subsequently at routine visits (1 month and 3 months post-transplantation) (25). After three months, graft survival will be evaluated to assess short-term complications; if the 3-months graft-survival is less than 80% the DHOPE-COR-NMP protocol will be considered unsuccessful. A follow-up period of three months after transplantation is chosen, as PNF and EAD typically occur early after transplantation (26). Although the primary study endpoint graft survival is set at 3 months, all transplant recipients will be followed for at least 12 months to monitor for potential late complications.

For peer review only - http://bmjopen.bmj.com/site/about/guidelines.xhtml

Study objective and endpoints

The study described in this protocol aims to increase the number of transplantable donor livers, by resuscitating and evaluating initially nationwide declined ECD livers through a combined protocol of DHOPE, COR and NMP, using an HBOC-based perfusion solution.

Primary study endpoint

The primary endpoint is graft survival at 3 months after transplantation, where graft survival is defined as the absence of retransplantation or patient death. Both overall graft survival and graft survival censored for patient death (decease of the patient with a functioning graft) will be calculated.

Secondary study endpoints

- Graft- and patient survival at 7 days and 3, 6 and 12 months.
- Primary non-function (PNF): absence of or minimal function of a liver graft after transplantation requiring re-transplantation or leading to patient death within 7 days after the procedure (27).
- Early allograft dysfunction (EAD), defined as one or more of the following criteria (28):
    - Recipient serum aspartate aminotransferase (AST) and/or alanine aminotransferase (ALT) >2000 IU/mL within the first 7 days after transplantation.
    - International normalized ratio (INR) of ≥1.6 on post-operative day 7
    - Recipient serum bilirubin levels of ≥171 μmol/L (10 mg/dL) on post-operative day 7.

- Development of clinically evident NAS (biliary strictures in presence of symptoms such as elevated cholestatic enzymes, jaundice, cholangitis or pruritus). The number of interventions, such as: endoscopic retrograde cholangiopancreatography (ERCP), percutaneous transhepatic cholangiography and drain (PTCD) and reoperation will also be recorded.
- Biochemical analysis of graft function and IRI determined by post-operative serum levels of ALT, AST, alkaline phosphatase (ALP), gamma-glutamyl transferase (γGT), INR, lactate dehydrogenase (LDH), creatinine, platelets and total bilirubin at postoperative day 0-7 and 1 and 3 months.

Donor and recipient in- and exclusion criteria

Adult patients (≥18 years old) on the UMCG waiting list for a liver transplantation, who gave informed consent for this study, will be included. In- and exclusion criteria are summarized in **Table 2**. Eligible patients will be asked for informed consent during the screening process for liver transplantation by a hepatologist or researcher who is well known with the topic and assigned to this task by the delegation log.

Donor risk factors precluding safe transplantation, such as concurrent donor malignancy and a fibrotic/cirrhotic macroscopic appearance of the liver are considered exclusion criteria for the study. Furthermore, an agonal phase of >2 hours in DCD donors is considered an exclusion criteria, as it is national policy for procurement teams to withdraw if circulatory arrest has not occurred within 2 hours after withdrawal of life support. However, formally long donor warm ischemia time is not an exclusion criterion for this study.

Study procedures

Allocation procedure

After the liver of a donor in the Netherlands is declined by all three centres for regular transplantation following static cold storage alone, it will again be offered to our centre for inclusion in this protocol. In compliance with ET rules, livers will be allocated only to patients who are identified in the ET match list, based on the ET allocation rules. If a liver is not accepted for a patient who has given informed consent for the DHOPE-COR-NMP trial, it will be allocated to another center in one of the ET member states, according to ET allocation rules. ET and the national competent authority, the Dutch Transplantation Foundation (Nederlandse Transplantatie Stichting) have agreed that this protocol does not interfere with the regular allocation rules within the Netherlands or ET.

Preparation of the liver

The donor liver will be procured by one of the national multi organ procurement teams. A standard surgical technique of *in situ* cold flush via the aorta with 4 - 7 L of University of Wisconsin (UW) cold storage solution (0 – 4°C), supplemented with 50.000 IU of heparin, will be used. If possible, the liver will be procured with a segment of 3-5 cm supratruncal aorta left attached to the coeliac trunk. The portal vein and common bile duct will be kept as long as possible. After procurement, the liver will be flushed via the portal vein with at least 1L of UW cold storage solution. The cystic duct will be ligated, and the bile duct will be gently flushed with Belzer UW cold storage solution. The liver will be kept on ice (static cold storage) during transport to the UMCG where cannulation of the supratruncal aorta, portal vein, and bile duct will be performed. Furthermore a small catheter will be inserted into the caval vein to collect venous perfusate samples.

For peer review only - http://bmjopen.bmj.com/site/about/guidelines.xhtml

Preparation of the perfusion solution

The perfusion solution containing HBOC-201 (Table 3) and taurocholic acid for continuous infusion will be prepared under sterile conditions. The perfusion fluid was developed by our research group and has been used successfully in a pre-clinical study and the first clinical patients (24,25). For the purpose of this study the perfusion fluid was evaluated and tested for stability and compatibility by the pharmacy of the UMCG. Sterile preparation of the perfusion fluid will be conducted by the pharmacy of the UMCG. Taurocholic acid will be bought from Sigma Aldrich (Saint Louis, USA) and subsequently prepared for clinical use by pharmacy A 15 (the Netherlands), according to good manufacturing practice (GMP). Taurocholic acid will be continuously infused into the perfusion solution at a rate of 7.7 mg/h from the start of the NMP phase. Furthermore, sodium-bicarbonate can be added to the perfusion solution to correct a low pH.

Perfusion device

The Liver Assist (Organ Assist, Groningen, the Netherlands) is a CE marked (European Union Certification of Safety, Health and Environmental Requirements) machine perfusion device for *ex situ* perfusion of donor livers. The Liver Assist enables perfusion of the liver via the portal vein and hepatic artery, using two centrifugal pumps to provide continuous and pulsatile flow, respectively. The system is pressure controlled, which provides auto regulation of the flow through the liver. The temperature can be set from 8 to 37 °C, and the preservation solution can be oxygenated by two hollow fiber membrane oxygenators.

Machine perfusion settings, viability assessment and subsequent transplantation

The Liver Assist will be primed with the HBOC-201-based perfusion solution. The machine perfusion protocol consists of 1h of DHOPE, followed by 1h of COR, and a minimum of 2.5 h

of NMP. During DHOPE, the fraction of inspired oxygen (FiO_2) will be set at a 100% and the O_2-flow at 1 L, as described previously (10). During COR and NMP the FiO_2 and O_2-flow will be adjusted according to arterial pO_2 and venous saturation at the level of the suprahepatic inferior vena cava, where arterial pO_2 should be 10.0 – 13.3 kPa and venous saturation 55-75%. During COR, the temperature and arterial and portal pressure will be gradually increased, as depicted in **Figure 2.**

During the first 2.5 h of NMP, viability assessment will be carried out. The liver should match all of the criteria as described in Table 1. These criteria were derived from literature and our own experience and reflect both hepatocellular and biliary viability (12,13,15,17,29). If the liver meets the predefined viability criteria, the recipient operation will be started. NMP of the liver will continue until recipient hepatectomy is nearly complete. The liver will then be disconnected from the machine and immediately flushed with 2 L cold Belzer UW cold storage solution to remove the HBOC-201-based perfusion fluid. If the liver does not meet the predefined viability criteria, the liver will be offered back to Eurotransplant for re-allocation or secondary discard.

Follow-up

After transplantation, patients will be monitored and treated according to standard post-transplant care. After discharge from the hospital, patients will be evaluated in the outpatient clinic up to 3 months. A summary of outcome parameters is shown in **Table 4**.

Statistics

A total of 10 liver transplantations will be included in this study to determine if the proposed protocol can be applied safely. To transplant 10 livers, we expect to assess 20 nationwide declined donor livers, as our preclinical study has indicated a potential recovery rate of at least 50% (30). The intervention group will not be compared to a control group, as the livers that will be used are initially nationwide declined livers and it would not be ethical to transplant these livers directly. It is our main concern that the livers are safely transplanted and can match post-operative outcomes of regularly transplanted livers. Graft survival at 3 months after transplantation was chosen as the primary endpoint, as described in the ' Methods section'. An acceptable graft survival would be at least >80%, based on data of regular transplantations (31). If the 3-month graft survival rate is <80%, the trial will be stopped. Primary and secondary outcomes are described under 'Study objective and endpoints'.

Collection, storage and analyses of data and samples

During machine perfusion and liver transplantation

- Perfusion characteristics, such as flow through the portal vein and hepatic artery, pressure settings for the portal vein and hepatic artery, resistance in the portal vein and hepatic artery, and temperature will be recorded every fifteen minutes.
- Every half hour arterial perfusate, and every hour venous perfusate, will be sampled for point of care analyses to evaluate liver function. Perfusate samples will be stored at -80 °C for later laboratory analyses, such as the assessment of hepatocellular injury markers and function.
- From COR/NMP onwards bile will be collected and stored at -80 °C for later assessment of biliary injury. Furthermore, every half hour bile samples will be

collected under mineral oil for point of care analysis to determine cholangiocyte function.

- Biopsies of liver parenchyma and common bile duct will be taken prior to machine perfusion, after machine perfusion and after reperfusion in the recipient. Biopsies will be snap frozen or stored in formalin for paraffin embedding. Hematoxylin and eosin (H&E) staining of paraffin biopsies will be performed to analyze hepatobiliary histological injury.
- Hemodynamic status (mean arterial pressure, heart rate and the use of inotropic medication) of the recipient will be recorded prior to, during and after graft reperfusion.

Other study parameters: Baseline values and parameters at inclusion

- Patient general demographics (age, gender, weight, height).
- Patient medical history, including:
    - Model for end-stage liver disease (MELD) score;
    - Indication for transplantation;
    - Symptoms of decompensated cirrhosis (i.e. ascites; encephalopathy; hepatorenal syndrome)
    - Viral status (i.e. hepatitis A – E; CMV; EBV)
    - Current hospitalization status (home, hospital ward or ICU);
    - Kidney function, as assessed by creatinine, glomerular filtration rate and the need for dialysis;
    - Medication.

- Donor and liver graft characteristics (age, gender, weight before and after machine perfusion, height, cause of death, viral status, liver function, amount of steatosis and donor risk index).
- Reason for initial decline of the liver graft.
- In case of DCD, characteristics such as time interval between withdrawal of life support and circulatory arrest, time interval between circulatory arrest and start cold perfusion *in situ,* donor hepatectomy time.
- Surgical methods and technical difficulties or abnormalities.
- Cold ischemia time, total preservation time (including SCS, DHOPE and NMP) and warm ischemia time during implantation.

Monitoring and reporting

Serious adverse events (SAE) will be reported to the accredited medical ethical committee through the national web portal *ToetsingOnline* within 15 days after detection. Monitor visits via the Trial Coordination Center (official institute for the monitoring of clinical trials in our centre) will take place after inclusion of 1-, 5-, and 10 patients. During these visits the monitor will examine the line listing of all adverse events.

Patient and public involvement.

Patients and/or public were not involved in the design of the study and the selection of outcome parameters. No patients are involved in the recruitment and/or conduct of the study. Dissemination of the results of the study will be via scientific congresses and meetings, publication(s) in peer-reviewed scientific journal(s), and may include a press release for lay

media. Participants in the trial will be informed about the outcomes through their local investigators.

DISCUSSION

ECD livers are increasingly accepted for transplantation, due to the persistent shortage of suitable donor livers, leading to waiting list morbidity and mortality. Although ECD livers are increasingly used, many livers are still declined for transplantation because the estimated risk of early graft loss after transplantation, such as PNF, EAD or biliary complications, is considered too high (1). The current study aims to increase the number of livers suitable for transplantation by resuscitating and evaluating initially nationwide declined ECD livers with a combined machine perfusion protocol, using an HBOC-based perfusion solution.

The risks associated with participation in this study are related to the use of ECD livers. However, current literature and data from our own research group suggest that the proposed protocol will be beneficial for such livers, leading to acceptable outcome after transplantation. DHOPE has been described to resuscitate mitochondria, increase ATP concentration, and reduce IRI of both the hepatocytes and cholangiocytes (8-10). Subsequently, COR prevents (mitochondrial) damage during and after a sudden temperature shift. Lastly, NMP will be used to assess viability (7,11).

The primary endpoint of this study is based on the increased risk of ECD livers to develop PNF, EAD and post-transplant cholangiopathy (1). As these complications mostly occur within 3 months, graft survival at 3 months after transplantation was chosen as the primary outcome (26). If graft survival is >80 %, we will consider the proposed protocol as feasible and safe. A graft survival of <80 % will be considered a cut-off point for this trial. These percentages are based on outcome of regular liver transplantations (31).

One of the down sides of using high-risk ECD liver that were initially nationwide declined is the inability to add a control group. In our opinion it would be unethical to transplant livers with a perceived high risk of complications, without resuscitating and evaluating these livers with machine perfusion prior to transplantation. For studies that include initially declined donor livers it is not unusual to not include a control group, as the

risk of developing severe complications would be too high. The VITTAL Trial has a similar design (18).

To combine the machine perfusion phases, DHOPE, COR and NMP an HBOC-201-based perfusion solution will be used. *In vivo* side effects of first generation HBOCs have been observed in the past. However, HBOC-201 recently received FDA-approval for application in selected centers to treat patients with life-threatening anemia, who are unable/unwilling to receive RBC (21,22). The use of HBOC-201 for machine perfusion is an *ex situ* application and three different groups have reported favorable preclinical results after machine perfusion with an HBOC-201-based perfusion solution (19,24,32). To mitigate the potential side effects of HBOC-201 liver grafts will be flushed thoroughly with regular Belzer static cold storage solution after the machine perfusion procedure.

In summary, with this protocol of sequential DHOPE, COR and NMP we aim to resuscitate and select initially declined suboptimal donor livers for transplantation, in order to increase the number of transplantable livers. The application of the combined machine perfusion protocol will be considered safe and feasible if graft survival at 3 months after transplantation of these initially declined donor livers is similar to that of regularly transplanted donor livers.

For peer review only - http://bmjopen.bmj.com/site/about/guidelines.xhtml

ETHICS AND DISSEMINATION

This study is investigator initiated and was approved by the medical ethical committee of Groningen, METc2016.281 in August 2016. Furthermore, approval was given by the Dutch Transplantation Society and the Eurotransplant organization to use initially nationwide declined donor livers for the proposed protocol. The device used for this study is CE marked, and its use was approved by the competent authority as well. As described in the Methods section the perfusion solution will be prepared by our hospital pharmacy according to good manufacturing practice, to ensure sterility and composition of the product. Monitoring of this study will be carried out by the trial coordination center of the UMCG after inclusion of 1, 5- and 10 transplantations to ensure compliance with the protocol and patient safety in this trial.

Patient data will be coded and can only be decrypted via a secured document. After 15 years, data will be destroyed. All team members involved with this trial will adhere to the code of good research conduct. The results of this study will be submitted to a peer reviewed international medical journal. Data of this study might be used for side studies.

For peer review only - http://bmjopen.bmj.com/site/about/guidelines.xhtml

REFERENCES

(1) Merion RM, Goodrich NP, Feng S. How can we define expanded criteria for liver donors? J Hepatol 2006 Oct;45(4):484-488.

(2) Orman ES, Mayorga ME, Wheeler SB, Townsley RM, Toro-Diaz HH, Hayashi PH, et al. Declining liver graft quality threatens the future of liver transplantation in the United States. Liver Transpl 2015 Aug;21(8):1040-1050.

(3) Orman ES, Barritt AS,4th, Wheeler SB, Hayashi PH. Declining liver utilization for transplantation in the United States and the impact of donation after cardiac death. Liver Transpl 2013 Jan;19(1):59-68.

(4) NTS jaarverslag 2016. 2018; Available at: https://www.transplantatiestichting.nl/sites/default/files/product/downloads/nts_jaarverslag_2016.pdf.

(5) op den Dries S, Karimian N, Sutton ME, Westerkamp AC, Nijsten MW, Gouw AS, et al. Ex vivo normothermic machine perfusion and viability testing of discarded human donor livers. Am J Transplant 2013 May;13(5):1327-1335.

(6) Schlegel A, Graf R, Clavien PA, Dutkowski P. Hypothermic oxygenated perfusion (HOPE) protects from biliary injury in a rodent model of DCD liver transplantation. J Hepatol 2013 Nov;59(5):984-991.

(7) Minor T, Efferz P, Fox M, Wohlschlaeger J, Luer B. Controlled oxygenated rewarming of cold stored liver grafts by thermally graduated machine perfusion prior to reperfusion. Am J Transplant 2013 Jun;13(6):1450-1460.

(8) Karangwa SA, Dutkowski P, Fontes P, Friend PJ, Guarrera JV, Markmann JF, et al. Machine Perfusion of Donor Livers for Transplantation: A Proposal for Standardized Nomenclature and Reporting Guidelines. Am J Transplant 2016 Oct;16(10):2932-2942.

(9) Schlegel A, Rougemont O, Graf R, Clavien PA, Dutkowski P. Protective mechanisms of end-ischemic cold machine perfusion in DCD liver grafts. J Hepatol 2013 Feb;58(2):278-286.

(10) van Rijn R, Karimian N, Matton APM, Burlage LC, Westerkamp AC, van den Berg, A P, et al. Dual hypothermic oxygenated machine perfusion in liver transplants donated after circulatory death. Br J Surg 2017 Jun;104(7):907-917

(11) Hoyer DP, Mathe Z, Gallinat A, Canbay AC, Treckmann JW, Rauen U, et al. Controlled Oxygenated Rewarming of Cold Stored Livers Prior to Transplantation: First Clinical Application of a New Concept. Transplantation 2016 Jan;100(1):147-152.

(12) Sutton ME, op den Dries S, Karimian N, Weeder PD, de Boer MT, Wiersema-Buist J, et al. Criteria for viability assessment of discarded human donor livers during ex vivo normothermic machine perfusion. PLoS One 2014 Nov 4;9(11):e110642.

(13) Watson CJ, Kosmoliaptsis V, Randle LV, Russell NK, Griffiths WJ, Davies S, et al. Preimplant Normothermic Liver Perfusion of a Suboptimal Liver Donated After Circulatory Death. Am J Transplant 2016 Jan;16(1):353-357.

(14) Watson CJE, Kosmoliaptsis V, Pley C, Randle L, Fear C, Crick K, et al. Observations on the ex situ perfusion of livers for transplantation. Am J Transplant 2018 Aug;18(8):2005-2020

(15) Weeder PD, van Rijn R, Porte RJ. Machine perfusion in liver transplantation as a tool to prevent non-anastomotic biliary strictures: Rationale, current evidence and future directions. J Hepatol 2015 Jul;63(1):265-275.

(16) Matton APM, de Vries Y, Burlage LC, van Rijn R, Fujiyoshi M, de Meijer VE, et al. Biliary Bicarbonate, pH and Glucose Are Suitable Biomarkers of Biliary Viability During Ex Situ Normothermic Machine Perfusion of Human Donor Livers. Transplantation 2018 November 01. doi: 10.1097/TP.0000000000002500.

(17) Mergental H, Perera M, Laing RW, Muiesan P, Isaac JR, Smith A, et al. Transplantation of Declined Liver Allografts Following Normothermic Ex-Situ Evaluation. Am J Transplant 2016 Nov;16(11):3235-3245.

(18) Laing RW, Mergental H, Yap C, Kirkham A, Whilku M, Barton D, et al. Viability testing and transplantation of marginal livers (VITTAL) using normothermic machine perfusion: study protocol for an open-label, non-randomised, prospective, single-arm trial. BMJ Open 2017 November 28;7(11):017733.

(19) Laing RW, Bhogal RH, Wallace L, Boteon Y, Neil DAH, Smith A, et al. The use of an Acellular Oxygen Carrier in a Human Liver Model of Normothermic Machine Perfusion. Transplantation 2017 Nov;101(11):2746-2756.

(20) Buttari B, Profumo E, Rigano R. Crosstalk between red blood cells and the immune system and its impact on atherosclerosis. Biomed Res Int 2015;2015:616834.

(21) Davis JM, El-Haj N, Shah NN, Schwartz G, Block M, Wall J, et al. Use of the blood substitute HBOC-201 in critically ill patients during sickle crisis: a three-case series. Transfusion 2018 Jan;58(1):132-137.

(22) Mer M, Hodgson E, Wallis L, Jacobson B, Levien L, Snyman J, et al. Hemoglobin glutamer-250 (bovine) in South Africa: consensus usage guidelines from clinician experts who have treated patients. Transfusion 2016 October 01;56(10):2631-2636.

(23) Fontes P, Lopez R, van der Plaats A, Vodovotz Y, Minervini M, Scott V, et al. Liver preservation with machine perfusion and a newly developed cell-free oxygen carrier solution under subnormothermic conditions. Am J Transplant 2015 Feb;15(2):381-394.

(24) Matton APM, Burlage LC, van Rijn R, de Vries Y, Karangwa SA, Nijsten MW, et al. Normothermic Machine Perfusion of Donor Livers Without the Need for Human Blood Products. Liver Transpl 2018 Apr;24(4):528-538

(25) de Vries Y, Matton APM, Nijsten MWN, Werner MJM, van den Berg, A P, de Boer MT, et al. Pretransplant sequential hypo- and normothermic machine perfusion of suboptimal livers donated after circulatory death using a hemoglobin-based oxygen carrier perfusion solution. Am J Transplant 2018 December 26. doi: 10.1111/ajt.15228.

(26) Verhoeven CJ, Farid WR, de Jonge J, Metselaar HJ, Kazemier G, van der Laan, L J. Biomarkers to assess graft quality during conventional and machine preservation in liver transplantation. J Hepatol 2014 Sep;61(3):672-684.

(27) Uemura T, Randall HB, Sanchez EQ, Ikegami T, Narasimhan G, McKenna GJ, et al. Liver retransplantation for primary nonfunction: analysis of a 20-year single-center experience. Liver Transpl 2007 Feb;13(2):227-233.

(28) Olthoff KM, Kulik L, Samstein B, Kaminski M, Abecassis M, Emond J, et al. Validation of a current definition of early allograft dysfunction in liver transplant recipients and analysis of risk factors. Liver Transpl 2010 Aug;16(8):943-949.

(29) Watson CJ, Kosmoliaptsis V, Randle LV, Gimson AE, Brais R, Klinck JR, et al. Normothermic perfusion in the assessment and preservation of declined livers prior to transplantation: hyperoxia and vasoplegia - important lessons from the first 12 cases. Transplantation 2017 Jan 25.

(30) Westerkamp AC, Karimian N, Matton AP, Mahboub P, van Rijn R, Wiersema-Buist J, et al. Oxygenated Hypothermic Machine Perfusion After Static Cold Storage Improves Hepatobiliary Function of Extended Criteria Donor Livers. Transplantation 2016 Apr;100(4):825-835.

(31) NTS jaarverslag 2015. Available at: http://www.transplantatiestichting.nl/sites/default/files/product/downloads/nts_jaarverslag_2015.pdf. Accessed April, 2017.

For peer review only - http://bmjopen.bmj.com/site/about/guidelines.xhtml

(32) Fontes P, Lopez R, van der Plaats A, Vodovotz Y, Minervini M, Scott V, et al. Liver preservation with machine perfusion and a newly developed cell-free oxygen carrier solution under subnormothermic conditions. Am J Transplant 2015 Feb;15(2):381-394.

LIST OF ABBREVIATONS

ALP	Alkaline phosphatase
ALT	Alanine aminotransferase
AST	Aspartate aminotransferase
ATP	Adenosine 5’-triphosphate
COR	Controlled oxygenated rewarming
DCD	Donation after circulatory death
DHOPE	Dual hypothermic oxygenated perfusion
EAD	Early allograft dysfunction
ECD	Extended criteria donor
ET	Eurotransplant
HBOC	Haemoglobin-based oxygen carrier
HMP	Hypothermic machine perfusion
INR	International normalized ratio
LDH	Lactate dehydrogenase
NAS	Non-anastomotic biliary strictures
NMP	Normothermic machine perfusion
PNF	Primary non-function
RBC	Red blood cells
SCS	Static cold storage
UMCG	University Medical Center Groningen
UW	University of Wisconsin
γGT	Gamma-glutamyl transferase

For peer review only - http://bmjopen.bmj.com/site/about/guidelines.xhtml

AUTHORS CONTRIBUTIONS

The study was initiated and designed by YdV and RJP. YdV, MF, APvdB, HB, MTdB, FvdH, RHJdK, OBvL, APMM, MJMW, TL, VEdM and RJP contributed to the final study design, which was determined during several meetings. The manuscript was drafted by YdV, TAB en RJP. YdV, TAB, MF, APvdB, HB, MTdB, FvdH, RHJdK, OBvL, APMM, MJMW, TL, VEdM and RJP edited the manuscript and approved the final version of the manuscript.

FUNDING STATEMENT

This work is supported by the Dutch Ministry of Health Welfare and Sport and the de Cock - Hadders Foundation, Groningen. The HBOC-201 is kindly provided by HBO_2 Therapeutics (Souderton, USA).

COMPETING INTEREST STATEMENT

The authors do have no conflict of interest to declare.

For peer review only - http://bmjopen.bmj.com/site/about/guidelines.xhtml

TABLES

Table 1. Viability criteria

- Cumulative bile production of ≥10 mL during first 150 min of NMP and ≥4 mL in the last hour (12).
- Lactate concentration in perfusate is [0.5 – 1.7 mmol/L] within 150 min of NMP (13,17).
- Perfusion fluid pH is [7.35 – 7.45] within 150 min of NMP, without the need for repeated addition of $NaHCO_3$ (13,17).
- Biliary pH of >7.45 within 150 minutes of NMP (15,29).

A liver graft will be deemed transplantable, if all of the criteria were met within 150 min of NMP. As described in the Introduction and in the Methods section, criteria were based on previous studies and own experience, and reflect both hepatocellular and biliary viability.

For peer review only - http://bmjopen.bmj.com/site/about/guidelines.xhtml

Table 2. In- and exclusion criteria.

	Inclusion	Exclusion
**Donor**	Body weight ≥40 kg	HIV, hepatitis B or C positive<br>Split or partial liver grafts<br>Domino donor livers<br>Expected CIT ≥10 hours [20]
**Recipient**	≥18 years old<br>Informed consent	HIV positive<br>Mental incapacitation<br>Fulminant liver failure or re-transplantation for PNF<br>Participation in another trial which might influence outcomes of this trial

Abbreviations: CIT, cold ischemia time; HIV, human immunodeficiency virus; PNF, primary non-function.

For peer review only - http://bmjopen.bmj.com/site/about/guidelines.xhtml

Table 3. Composition of the HBOC-201 based perfusion solution.

Component	Manufacturer/Distributor	Volume (mL)
HBOC-201 (Hemopure)	HbO_2 Therapeutics LCC, PA	1250
Gelofusine 4%	B Braun, Melsungen, Germany	300
Albumin 20%	Sanquin® (Dutch blood bank)	250
Total parenteral nutrition (TPN N14G30E)	Hospital pharmacy	20
Addamel (trace elements)	Fresenius Kabi, Netherlands	10
Metronidazol (Flagyl) 5 mg/ml	Baxter BV, Utrecht, Netherlands	44
Sterile water	B Braun, Melsungen, Germany	335
Insulin (NovoRapid) 100 IU/ml	Novo Nordisk BV, Alphen aan den Rijn, Netherlands	1
Cernevit (Multi vitamins)	Baxter BV, Utrecht, Netherlands	2
Heparin (5000 IU/ml)	Leo Pharma, Amsterdam Netherlands	2
Cefazolin 1g /5mL	Baxter BV, Utrecht, Netherlands	2
Taurocholic acid sodium salt 0.1% (1mg/mL)	Sigma Aldrich, Saint Louis, USA	7,7
Sodium bicarbonate 8.4%	B Braun, Melsungen, Germany	35
KCl 1mmol/mL	B Braun, Melsungen, Germany	2
Glutathion (Tationil) 600mg/4mL	Teofarma, Pavia, Italy	14
Total volume		2274,7

For peer review only - http://bmjopen.bmj.com/site/about/guidelines.xhtml

Table 4. Laboratory investigations during follow-up.

Aspartate aminotransferase
Alanine aminotransferase
Alkaline phosphatase
Gamma-glutamyl transferase
Total bilirubin
Lactate dehydrogenase
Creatinine
Platelets
International normalized ratio

FIGURES LEGENDS

Figure 1. Timeline of a regular liver transplantation vs. the described protocol. The colored bar depicts the machine perfusion protocol. The light blue bar represents 1h of DHOPE, the light orange bar represents 1h of COR and the pink bar represents NMP. If the liver is deemed transplantable within 150 minutes of NMP, NMP will continue (indicated in pink). Note that SCS, and transport are approximate. Cold ischemia time (CIT) is defined as the time from the start of cold *in situ* flush in the donor until reperfusion in the recipient. In the proposed protocol CIT is defined as the time from the start of cold flush *in situ* until the start of machine perfusion. Abbreviations: SCS, static cold storage.

Figure 2. Liver Assist pressure and temperature settings during DHOPE, COR and NMP. Abbreviations: COR, controlled oxygenated rewarming; DHOPE, dual hypothermic oxygenated perfusion; HA, hepatic artery; NMP, normothermic machine perfusion; PV, portal vein.

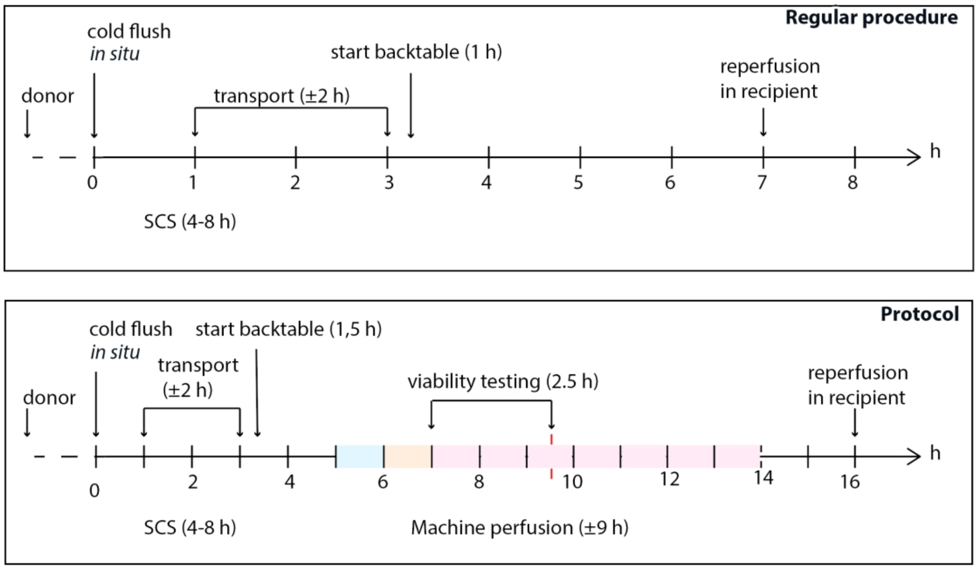
Regular procedure
cold flush
in situ
start backtable (1 h)
reperfusion
in recipient
donor
transport (±2 h)
h
0
1
2
3
4
5
6
7
8
SCS (4-8 h)
Protocol
cold flush
in situ
start backtable (1,5 h)
transport
(±2 h)
viability testing (2.5 h)
reperfusion
in recipient
donor
h
0
2
4
6
8
10
12
14
16
SCS (4-8 h)
Machine perfusion (±9 h)

Figure 1

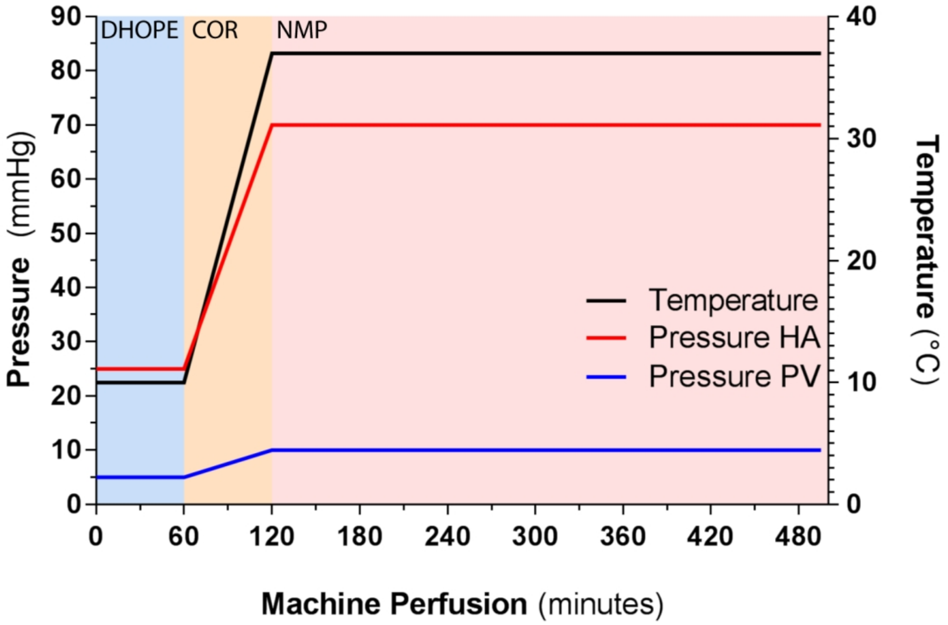
DHOPE
COR
NMP
Pressure (mmHg)
Temperature (°C)
Temperature
Pressure HA
Pressure PV
Machine Perfusion (minutes)
0
10
20
30
40
50
60
70
80
90
0
60
120
180
240
300
360
420
480

Figure 2

SPIRIT

STANDARD PROTOCOL ITEMS: RECOMMENDATIONS FOR INTERVENTIONAL TRIALS

SPIRIT 2013 Checklist: Recommended items to address in a clinical trial protocol and related documents*

Section/item	Item No	Description	
**Administrative information**			
Title	1	Descriptive title identifying the study design, population, interventions, and, if applicable, trial acronym	✓
Trial registration	2a	Trial identifier and registry name. If not yet registered, name of intended registry	✓ page6
	2b	All items from the World Health Organization Trial Registration Data Set	NA
Protocol version	3	Date and version identifier	
Funding	4	Sources and types of financial, material, and other support	✓ page 25
Roles and responsibilities	5a	Names, affiliations, and roles of protocol contributors	✓ page 1 + 23
	5b	Name and contact information for the trial sponsor	✓ page 1
	5c	Role of study sponsor and funders, if any, in study design; collection, management, analysis, and interpretation of data; writing of the report; and the decision to submit the report for publication, including whether they will have ultimate authority over any of these activities	Page 25
	5d	Composition, roles, and responsibilities of the coordinating centre, steering committee, endpoint adjudication committee, data management team, and other individuals or groups overseeing the trial, if applicable (see Item 21a for data monitoring committee)	N.A. for monitoring see page 18
**Introduction**			
Background and rationale	6a	Description of research question and justification for undertaking the trial, including summary of relevant studies (published and unpublished) examining benefits and harms for each intervention	✓ page 5-8
	6b	Explanation for choice of comparators	N.A.
Objectives	7	Specific objectives or hypotheses	✓ page 10
Trial design	8	Description of trial design including type of trial (eg, parallel group, crossover, factorial, single group), allocation ratio, and framework (eg, superiority, equivalence, noninferiority, exploratory)	✓ page 9

For peer review only - http://bmjopen.bmj.com/site/about/guidelines.xhtml

Allocation concealment mechanism	16b	Mechanism of implementing the allocation sequence (eg, central telephone; sequentially numbered, opaque, sealed envelopes), describing any steps to conceal the sequence until interventions are assigned NA
Implementation	16c	Who will generate the allocation sequence, who will enrol participants, and who will assign participants to interventions NA
Blinding (masking)	17a	Who will be blinded after assignment to interventions (eg, trial participants, care providers, outcome assessors, data analysts), and how NA
	17b	If blinded, circumstances under which unblinding is permissible, and procedure for revealing a participant's allocated intervention during the trial NA.

Methods: Data collection, management, and analysis

Data collection methods	18a	Plans for assessment and collection of outcome, baseline, and other trial data, including any related processes to promote data quality (eg, duplicate measurements, training of assessors) and a description of study instruments (eg, questionnaires, laboratory tests) along with their reliability and validity, if known. Reference to where data collection forms can be found, if not in the protocol page 16&17.
	18b	Plans to promote participant retention and complete follow-up, including list of any outcome data to be collected for participants who discontinue or deviate from intervention protocols N.A.
Data management	19	Plans for data entry, coding, security, and storage, including any related processes to promote data quality (eg, double data entry; range checks for data values). Reference to where details of data management procedures can be found, if not in the protocol page 21
Statistical methods	20a	Statistical methods for analysing primary and secondary outcomes. Reference to where other details of the statistical analysis plan can be found, if not in the protocol page 14
	20b	Methods for any additional analyses (eg, subgroup and adjusted analyses) N.A.
	20c	Definition of analysis population relating to protocol non-adherence (eg, as randomised analysis), and any statistical methods to handle missing data (eg, multiple imputation) NA.

Methods: Monitoring

Data monitoring	21a	Composition of data monitoring committee (DMC); summary of its role and reporting structure; statement of whether it is independent from the sponsor and competing interests; and reference to where further details about its charter can be found, if not in the protocol. Alternatively, an explanation of why a DMC is not needed N.A. page 18

Appendices

Informed consent materials	32	Model consent form and other related documentation given to participants and authorised surrogates seperate from protocol, not included
Biological specimens	33	Plans for collection, laboratory evaluation, and storage of biological specimens for genetic or molecular analysis in the current trial and for future use in ancillary studies, if applicable page 21.

*It is strongly recommended that this checklist be read in conjunction with the SPIRIT 2013 Explanation & Elaboration for important clarification on the items. Amendments to the protocol should be tracked and dated. The SPIRIT checklist is copyrighted by the SPIRIT Group under the Creative Commons "Attribution-NonCommercial-NoDerivs 3.0 Unported" license.

For peer review only - http://bmjopen.bmj.com/site/about/guidelines.xhtml

Methods: Participants, interventions, and outcomes

Study setting	9	Description of study settings (eg, community clinic, academic hospital) and list of countries where data will be collected. Reference to where list of study sites can be obtained NA.
Eligibility criteria	10	Inclusion and exclusion criteria for participants. If applicable, eligibility criteria for study centres and individuals who will perform the interventions (eg, surgeons, psychotherapists) √page 11
Interventions	11a	Interventions for each group with sufficient detail to allow replication, including how and when they will be administered one group √ page 11 - 14
	11b	Criteria for discontinuing or modifying allocated interventions for a given trial participant (eg, drug dose change in response to harms, participant request, or improving/worsening disease) NA.
	11c	Strategies to improve adherence to intervention protocols, and any procedures for monitoring adherence (eg, drug tablet return, laboratory tests) N. A.
	11d	Relevant concomitant care and interventions that are permitted or prohibited during the trial NA.
Outcomes	12	Primary, secondary, and other outcomes, including the specific measurement variable (eg, systolic blood pressure), analysis metric (eg, change from baseline, final value, time to event), method of aggregation (eg, median, proportion), and time point for each outcome. Explanation of the clinical relevance of chosen efficacy and harm outcomes is strongly recommended √page 10+11 √page 15+16 table 4.
Participant timeline	13	Time schedule of enrolment, interventions (including any run-ins and washouts), assessments, and visits for participants. A schematic diagram is highly recommended (see Figure) √page 14 see point 12
Sample size	14	Estimated number of participants needed to achieve study objectives and how it was determined, including clinical and statistical assumptions supporting any sample size calculations √page 14
Recruitment	15	Strategies for achieving adequate participant enrolment to reach target sample size √ page 11

Methods: Assignment of interventions (for controlled trials)

Allocation:

Sequence generation	16a	Method of generating the allocation sequence (eg, computer-generated random numbers), and list of any factors for stratification. To reduce predictability of a random sequence, details of any planned restriction (eg, blocking) should be provided in a separate document that is unavailable to those who enrol participants or assign interventions NA.

For peer review only - http://bmjopen.bmj.com/site/about/guidelines.xhtml

	21b	Description of any interim analyses and stopping guidelines, including who will have access to these interim results and make the final decision to terminate the trial NA. page 14
Harms	22	Plans for collecting, assessing, reporting, and managing solicited and spontaneously reported adverse events and other unintended effects of trial interventions or trial conduct page 18
Auditing	23	Frequency and procedures for auditing trial conduct, if any, and whether the process will be independent from investigators and the sponsor N.A.

Ethics and dissemination

Research ethics approval	24	Plans for seeking research ethics committee/institutional review board (REC/IRB) approval page 21.
Protocol amendments	25	Plans for communicating important protocol modifications (eg, changes to eligibility criteria, outcomes, analyses) to relevant parties (eg, investigators, REC/IRBs, trial participants, trial registries, journals, regulators) page 21.
Consent or assent	26a	Who will obtain informed consent or assent from potential trial participants or authorised surrogates, and how (see Item 32) page 11
	26b	Additional consent provisions for collection and use of participant data and biological specimens in ancillary studies, if applicable N.A.
Confidentiality	27	How personal information about potential and enrolled participants will be collected, shared, and maintained in order to protect confidentiality before, during, and after the trial page 21
Declaration of interests	28	Financial and other competing interests for principal investigators for the overall trial and each study site page 25
Access to data	29	Statement of who will have access to the final trial dataset, and disclosure of contractual agreements that limit such access for investigators page 21
Ancillary and post-trial care	30	Provisions, if any, for ancillary and post-trial care, and for compensation to those who suffer harm from trial participation insurance sheet for patients not includen in protocol for publication
Dissemination policy	31a	Plans for investigators and sponsor to communicate trial results to participants, healthcare professionals, the public, and other relevant groups (eg, via publication, reporting in results databases, or other data sharing arrangements), including any publication restrictions page 21.
	31b	Authorship eligibility guidelines and any intended use of professional writers N.A.
	31c	Plans, if any, for granting public access to the full protocol, participant-level dataset, and statistical code N.A.
